# A Next-Generation Cleaved, Soluble HIV-1 Env Trimer, BG505 SOSIP.664 gp140, Expresses Multiple Epitopes for Broadly Neutralizing but Not Non-Neutralizing Antibodies

**DOI:** 10.1371/journal.ppat.1003618

**Published:** 2013-09-19

**Authors:** Rogier W. Sanders, Ronald Derking, Albert Cupo, Jean-Philippe Julien, Anila Yasmeen, Natalia de Val, Helen J. Kim, Claudia Blattner, Alba Torrents de la Peña, Jacob Korzun, Michael Golabek, Kevin de los Reyes, Thomas J. Ketas, Marit J. van Gils, C. Richter King, Ian A. Wilson, Andrew B. Ward, P. J. Klasse, John P. Moore

**Affiliations:** 1 Department of Microbiology and Immunology, Weill Medical College of Cornell University, New York, New York, United States of America; 2 Department of Medical Microbiology, Academic Medical Center, University of Amsterdam, Amsterdam, The Netherlands; 3 Department of Integrative Structural and Computational Biology, IAVI Neutralizing Antibody Center and CHAVI-ID, The Scripps Research Institute, La Jolla, California, United States of America; 4 International AIDS Vaccine Initiative, New York, New York, United States of America; 5 The Skaggs Institute for Chemical Biology, The Scripps Research Institute, La Jolla, California, United States of America; University of Zurich, Switzerland

## Abstract

A desirable but as yet unachieved property of a human immunodeficiency virus type 1 (HIV-1) vaccine candidate is the ability to induce broadly neutralizing antibodies (bNAbs). One approach to the problem is to create trimeric mimics of the native envelope glycoprotein (Env) spike that expose as many bNAb epitopes as possible, while occluding those for non-neutralizing antibodies (non-NAbs). Here, we describe the design and properties of soluble, cleaved SOSIP.664 gp140 trimers based on the subtype A transmitted/founder strain, BG505. These trimers are highly stable, more so even than the corresponding gp120 monomer, as judged by differential scanning calorimetry. They are also homogenous and closely resemble native virus spikes when visualized by negative stain electron microscopy (EM). We used several techniques, including ELISA and surface plasmon resonance (SPR), to determine the relationship between the ability of monoclonal antibodies (MAbs) to bind the soluble trimers and neutralize the corresponding virus. In general, the concordance was excellent, in that virtually all bNAbs against multiple neutralizing epitopes on HIV-1 Env were highly reactive with the BG505 SOSIP.664 gp140 trimers, including quaternary epitopes (CH01, PG9, PG16 and PGT145). Conversely, non-NAbs to the CD4-binding site, CD4-induced epitopes or gp41_ECTO_ did not react with the trimers, even when their epitopes were present on simpler forms of Env (e.g. gp120 monomers or dissociated gp41 subunits). Three non-neutralizing MAbs to V3 epitopes did, however, react strongly with the trimers but only by ELISA, and not at all by SPR and to only a limited extent by EM. These new soluble trimers are useful for structural studies and are being assessed for their performance as immunogens.

## Author Summary

A protective HIV-1 vaccine is badly needed, but no candidate has yet provided an adequate level of protection against infection. Most existing vaccines provide immune protection by inducing neutralizing antibodies, also a goal of many HIV-1 immunogen design projects. The trimeric envelope protein complex on the HIV-1 surface is the only relevant target for neutralizing antibodies, and is the basis for most strategies aimed at their induction. However, making a soluble, recombinant envelope protein complex that adequately mimics the structure present on the virus has been challenging. Here, we describe a newly designed and engineered Env protein that has the appropriate properties. This protein, termed BG505 SOSIP.664 gp140, binds most of the known neutralizing antibodies but generally does not bind antibodies that lack neutralization activity. Its appearance in negative stain electron micrographs also resembles native envelope complexes.

## Introduction

One approach to creating a preventative vaccine against human immunodeficiency virus type 1 (HIV-1) infection is to design an immunogen capable of inducing adequate titers of broadly neutralizing antibodies (bNAbs) [1]. NAbs prevent HIV-1 from infecting target cells by binding to the viral envelope glycoprotein (Env) complex, a trimeric structure comprising three gp120 and three gp41 subunits held together by meta-stable, non-covalent interactions. Induction of NAbs therefore requires the use of an Env-based immunogen. Of these, the most widely tested have been monomeric gp120 subunits, which failed to induce bNAbs and did not prevent infection [2], [3], [4]. A better mimic of the native, trimeric Env spike may be a superior immunogen for bNAb induction [1], [5], [6], [7], [8]. However, creating a true mimic of an Env trimeric spike has proven challenging.

Most approaches to making Env trimers involve truncating the gp41 component to remove the hydrophobic transmembrane region, yielding soluble gp140 proteins containing three gp120 and gp41 ectodomain (gp41_ECTO_) subunits [9]. Soluble gp140 trimers are highly unstable, perhaps because the inherently labile nature of the Env complex is exacerbated by the removal of the transmembrane region. Accordingly, gp140 trimers rapidly disintegrate into individual gp120 and gp41_ECTO_ subunits unless preventative steps are taken. Two different methods have been used to stabilize gp140 trimers. The most widely used involves eliminating the cleavage site between gp120 and gp41_ECTO_ and, in some cases, adding an additional trimer-stabilizing motif to the C-terminus of gp41_ECTO_, with or without other modifications [9], [10], [11], [12], [13], [14], [15], [16], [17], [18], [19]. Trimer-forming constructs such as these are generally referred to as uncleaved gp140s (gp140_UNC_). Our alternative approach involves making fully cleaved trimers but stabilizing them by introducing specific mutations, namely a disulfide bond to covalently link gp120 to gp41_ECTO_ and an Ile/Pro change at residue 559 to strengthen interactions between the gp41 subunits [5], [6]. The resulting trimers are designated SOSIP gp140s. Cleaved and uncleaved trimers are known to be antigenically distinct, in that the latter consistently express the epitopes for various non-neutralizing antibodies (non-NAbs) that are occluded on cleaved trimers, irrespective of whether the Env proteins are soluble or expressed on the cell surface [5], [20], [21], [22].

Here, we describe a new version of SOSIP gp140 trimers based on the subtype A transmitted/founder (T/F) virus sequence BG505, modified to introduce some bNAb epitopes. The membrane-proximal external region (MPER) was also deleted to improve trimer solubility and reduce aggregate formation [23], [24]. The BG505 SOSIP.664 gp140 trimers can be produced efficiently and are homogenous and stable. We show here that they express the epitopes for multiple bNAbs, but very few for non-neutralizing antibodies (non-NAbs), when analyzed by ELISA, surface plasmon resonance (SPR), isothermal calorimetry (ITC) and negative stain electron microscopy (EM). Their antigenic properties mimic those of the native Env complexes on the BG505 virus, as judged by the outcome of virus-neutralization assays, and they structurally resemble the native complexes when viewed by negative stain EM. These new trimers are the basis for a range of studies of Env structure, alone and as complexes with bNAbs. They have already been used to characterize the epitopes for several bNAbs, including PG9, PGT122 and PGT135 [25], [26], [27]. The BG505 SOSIP.664 trimers may also be useful as immunogens.

## Results

### Design of BG505 SOSIP.664 gp140 trimers

Here, we describe the production and properties of stable and homogenous SOSIP gp140 trimers that express multiple bNAb epitopes, based on the BG505 *env* gene. HIV-1 BG505, a subtype A T/F virus, was isolated from an infant 6-weeks after birth in a mother-infant transmission study [28]. The *env* sequence was then selected *in silico* based on its similarity to sequences in the individual from whom the bNAbs PG9 and PG16 were isolated [29]. The monomeric BG505 gp120 protein has the unusual property of binding PG9, although it does not bind efficiently to PG16, and not at all to PGT145; the latter two bNAbs appear to be more dependent on the quaternary structure of the Env trimer [27], [29].

**Figure 1:**
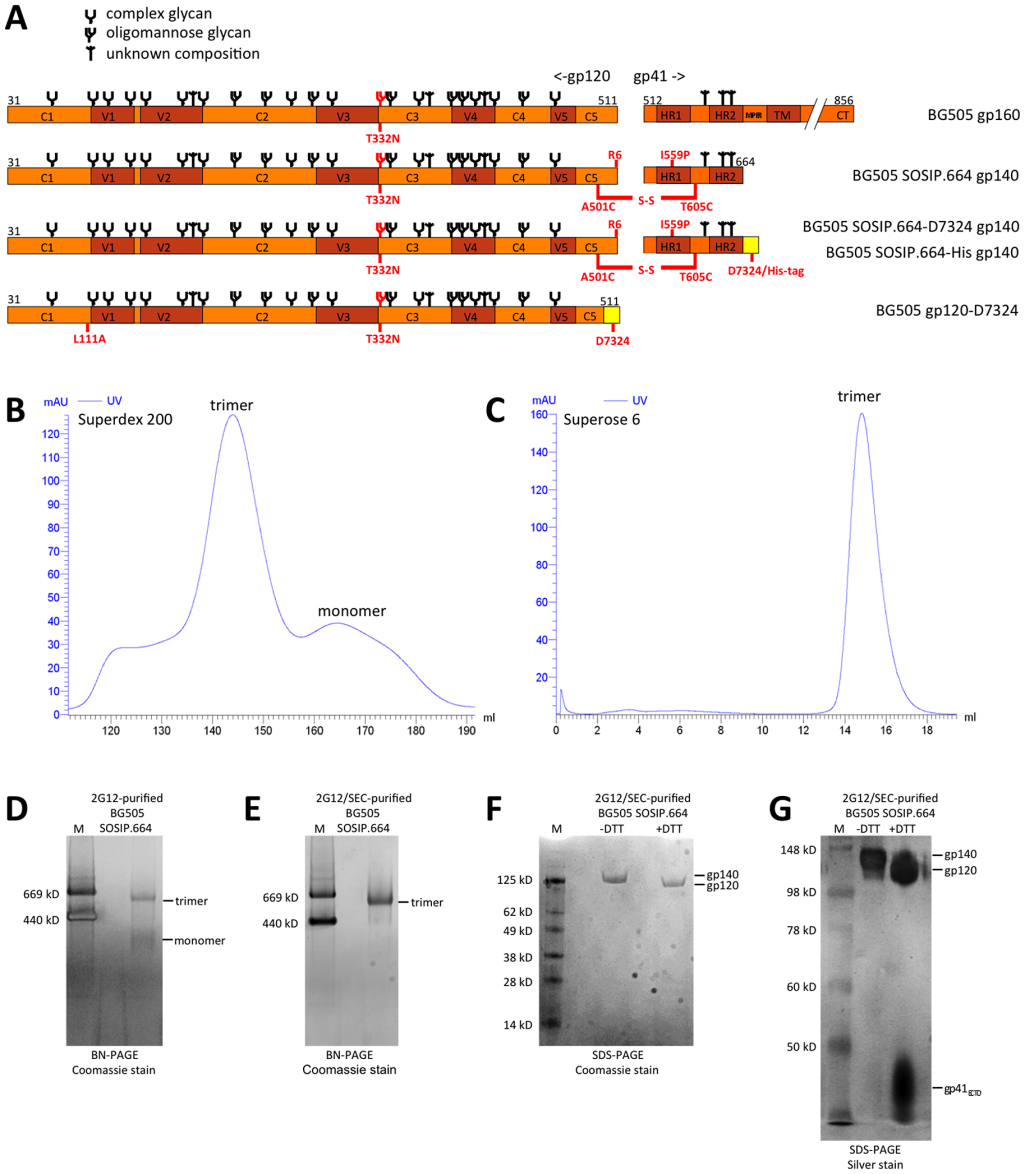
Design and biochemical characterization of BG505 SOSIP.664 gp140 trimers. (**A**) Linear representation of the BG505 gp160, SOSIP.664 gp140, SOSIP.664-D7324 gp140 and gp120-D7324 Env proteins. Modifications compared to the original BG505 gp160 sequence are indicated in red and mentioned in the text. The following changes were made to the wild type BG505 amino acid sequence: 1) The tissue plasminogen activator (tPA) signal peptide replaced the natural one; 2) the gp41 transmembrane (TM) and cytoplasmic tail (CT) domains were deleted to create a soluble gp140; 3) the A501C and T605C substitutions were made to form a disulfide bond between gp120 and gp41_ECTO_ [5]; 4) the I559P substitution was included to promote trimerization [6], [80]; 5) an optimal cleavage site (RRRRRR; R6) replaces the natural one, REKR [31]; 6) truncation of the MPER from residue-664 prevents aggregation [23], [24]; 7) the T332N substitution facilitates binding of bNAbs dependent on glycan-N332. The D7324- and His-tags are indicated in yellow. Env sub-domains are indicated: 5 conserved domains (C1–C5); 5 variable domains (V1–V5); heptad repeats 1 and 2 (HR1, HR2); the membrane proximal external region (MPER); the transmembrane domain (TM); and the cytoplasmic tail (CT). The glycan assignments in Env are based on previous studies using gp120 [81], [82], [83], but may be different for trimeric Env [84]. The amino acid sequence of BG505 SOSIP.664 is given in Fig. S1. (**B**) SEC profile of 2G12-purified BG505 SOSIP.664 gp140 expressed in CHO-K1 cells. A Superdex 200 26/60 column was used. (**C**) Analytical SEC profile of 2G12/SEC-purified BG505 SOSIP.664 trimer re-run on a Superose 6 10/30 column. (**D**) BN-PAGE analysis of CHO-K1 expressed, 2G12-purified BG505 SOSIP.664 gp140, stained by Coomassie blue. The m.w. of marker (M) proteins (thyroglobulin and ferritin) are indicated. (**E**) BN-PAGE analysis of 2G12/SEC-purified BG505 SOSIP.664 gp140, stained by Coomassie blue. (**F**) SDS-PAGE analysis using a 4–12% Bis-Tris Nu-PAGE gel of 2G12/SEC-purified BG505 SOSIP.664 gp140, under non-reducing and reducing conditions, followed by Coomassie blue staining. (**G**) SDS-PAGE analysis using a 10% Tris-Glycine gel of 2G12/SEC-purified BG505 SOSIP.664 gp140, under non-reducing and reducing conditions, followed by silver staining. The conversion of the gp140 band to gp120 and the appearance of a gp41_ECTO_ band under reducing conditions is indicative of cleavage.

Various sequence modifications (see Methods and legend to Fig. 1A) were made to the wild type BG505 sequence to create the protein designated BG505 SOSIP.664 gp140 (Fig. 1A). These alterations included the SOS and I559P changes required for trimer stability, the deletion of the MPER to improve homogeneity and solubility, and the introduction of a T332N substitution to create the epitopes for several bNAbs that depend on the presence of this glycan [5], [6], [24], [30], [31]. We also generated two variants with either a D7324-epitope tag or a His-tag located immediately downstream from residue 664, to permit the oriented immobilization of trimers on ELISA plates or SPR chips [32], [33], [34]. These proteins are designated SOSIP.664-D7324 gp140 and SOSIP.664-His gp140, respectively (Fig. 1A). For comparison, we expressed and purified a monomeric BG505 gp120 protein containing the same T332N knock-in substitution made to the SOSIP.664 gp140 trimer, as well as the D7324 epitope introduced into the C5 region (see Methods) (Fig. 1A). The BG505 WT.664-His gp140 construct serves as a source of gp41_ECTO_-His for ELISA studies (see Methods).

### Biochemical and biophysical characterization of BG505 SOSIP.664 gp140 trimers

The BG505 SOSIP.664 construct was expressed transiently in HEK293T, or in some experiments CHO-K1, cells together with co-transfected Furin to boost the level of cleavage [5], [31]. The two cell substrates yielded trimers of similar quality and antigenicity. The secreted Env proteins were affinity-purified using the 2G12 bNAb, followed by SEC on a Superdex 200 26/60 column to isolate trimers (Fig. 1B). The SEC profile showed that a predominant, trimer-containing peak eluted at 144 ml, while a smaller peak at 164 ml contained SOSIP gp140 monomers. The SEC profile was confirmed by a BN-PAGE analysis followed by Coomassie Blue dye staining; trimers predominated and some gp140 monomers were present, but there were no appreciable amounts of dimers or higher m.wt. aggregates (Fig. 1D). An analytical Superose 6 column assessment of the SEC-purified BG505 SOSIP.664 gp140 trimers showed that they remained trimeric and neither aggregated nor dissociated into gp120 or SOSIP gp140 monomers (Fig. 1C). These results were confirmed by BN-PAGE (Fig. 1E). The lack of aggregates most likely reflects the beneficial effect of deleting the MPER to make the SOSIP.664 variant [23], [24]. A single gp140 band was seen on an SDS-PAGE gel performed under non-reducing conditions, with no evidence for the formation of aberrant inter-protomer disulfide-bonds (Fig. 1F). Coomassie blue- or silver-stained SDS-PAGE gels showed that the gp140 band was essentially fully converted (>95%) to gp120 and gp41_ECTO_ when a reducing agent was present, confirming that the trimers were cleaved efficiently (Fig. 1F,G). Western blotting with anti-gp120 and anti-gp41 MAbs yielded a similar conclusion (data not shown).

**Figure 2:**
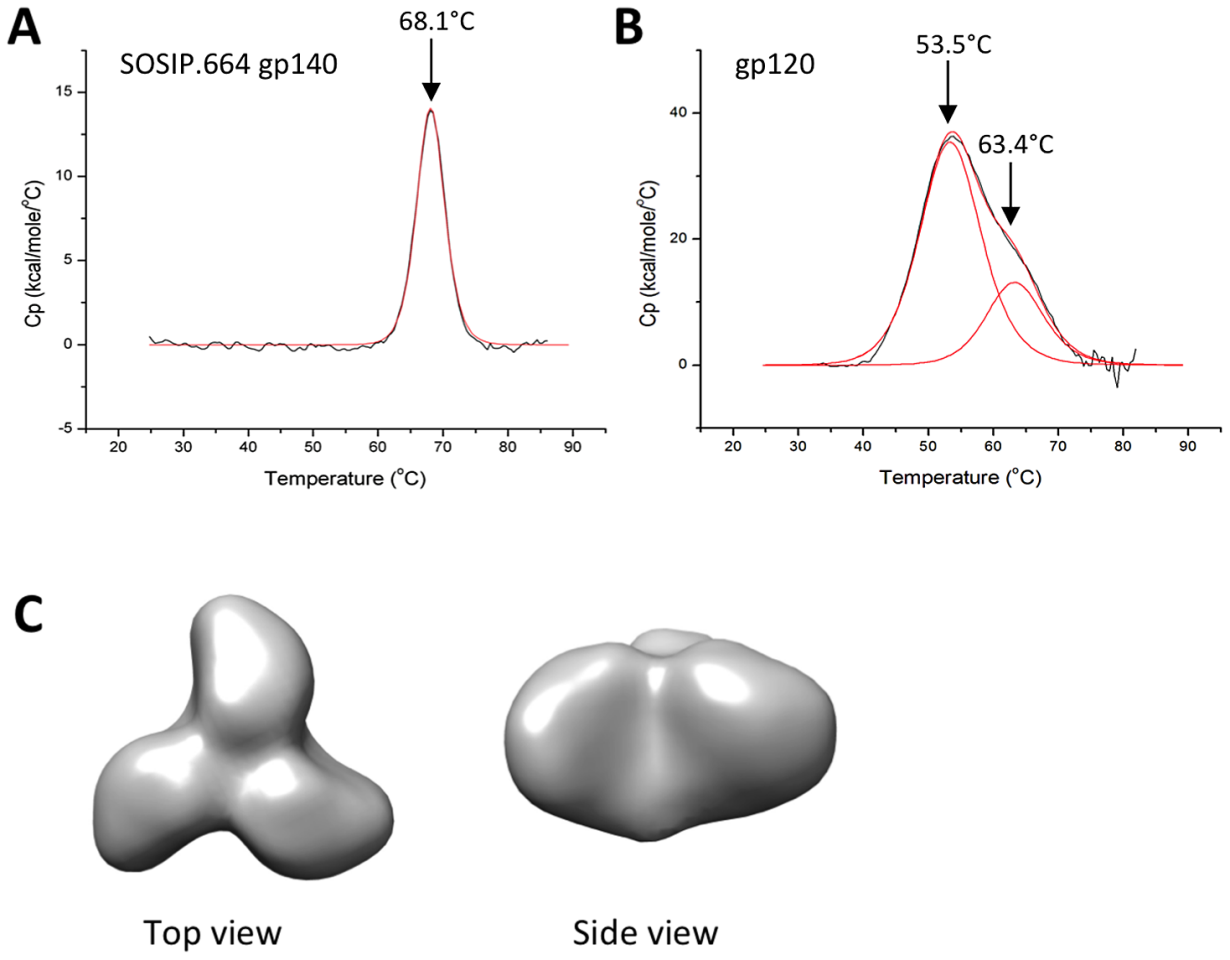
Biophysical characterization of BG505 SOSIP.664 gp140 trimers. DSC analysis of (**A**) purified BG505 SOSIP.664 gp140 trimers and (**B**) purified BG505 gp120 monomers. The melting profiles show that the trimer has a higher degree of stability than its monomeric counterpart, as its thermal transitions are initiated at a 14.4°C higher temperature. Raw data are shown in black, while the fitted curves from which T_m_ values were obtained are in red. (**C**) EM reconstruction of the BG505 SOSIP.664 gp140 trimer at 24-Å resolution. The 2D class averages and a Fourier Shell Correlation (FSC) curve are shown in Fig. S2.

We used differential scanning calorimetry (DSC) to assess the thermal stability of the purified, HEK293T cell-expressed BG505 SOSIP.664 gp140 trimers. The DSC profile showed one distinct unfolding peak with a thermal denaturation midpoint (T_m_) of 68.1°C (Fig. 2A). This finding was similar to ones made previously using the same trimers, but produced in HEK293S cells defective for GlcNAc transferase I (GnTI) and, hence, bearing only oligomannose glycans [27]. Of note is that the BG505 SOSIP.664 gp140 trimers are substantially more stable than the corresponding gp120 monomers, which unfold in two phases (T_m_, 53.5°C and 63.4°C; Fig. 2B), and also than YU2 gp120 (T_m_, 59.2°C [35], [36]) or 92UG031 gp120 (T_m_, 58.4°C [35]). The BG505 SOSIP.664 trimers are also more stable than the corresponding trimers from KNH1144 (T_m_ of 51.3°C for the first thermal transition [27]), and than JR-FL SOSIP.R6 trimers (which dissociate at ∼50°C [6]). We conclude that the BG505 SOSIP.664 gp140 trimers have high thermal stability.

The overall morphology of SEC-purified, BG505 SOSIP.664 gp140 trimers was studied by negative stain EM (Fig. 2C; Fig. S1). A 3D reconstruction at 24 Å resolution showed that compact and homogeneous trimers were consistently present, as described previously for the same trimers produced in HEK293S cells [27]. We obtained similar results with the SOSIP.664-D7324 and SOSIP.664-His gp140 proteins, indicating that the C-terminal tags did not perturb the overall trimer structure (data not shown). Additional EM images of trimer-bNAb complexes are described below.

### Neutralization of the parental BG505.T332N virus

To study the antigenic properties of the BG505 SOSIP.664 gp140 trimers, we first tested a large panel of MAbs for their capacity to neutralize the corresponding Env-pseudotyped virus in a TZM-bl cell-based neutralization assay. The epitope clusters recognized by the MAbs cover most of the surface of the Env trimer [37] except the MPER, which was truncated in the SOSIP.664 construct. Note that the test virus contains the T332N knock-in change to allow comparison with antigenicity data obtained using the BG505 SOSIP.664 gp140 trimers; this change may account for any discrepancies from data described elsewhere using the unmodified BG505 virus [29].

**Figure 3:**
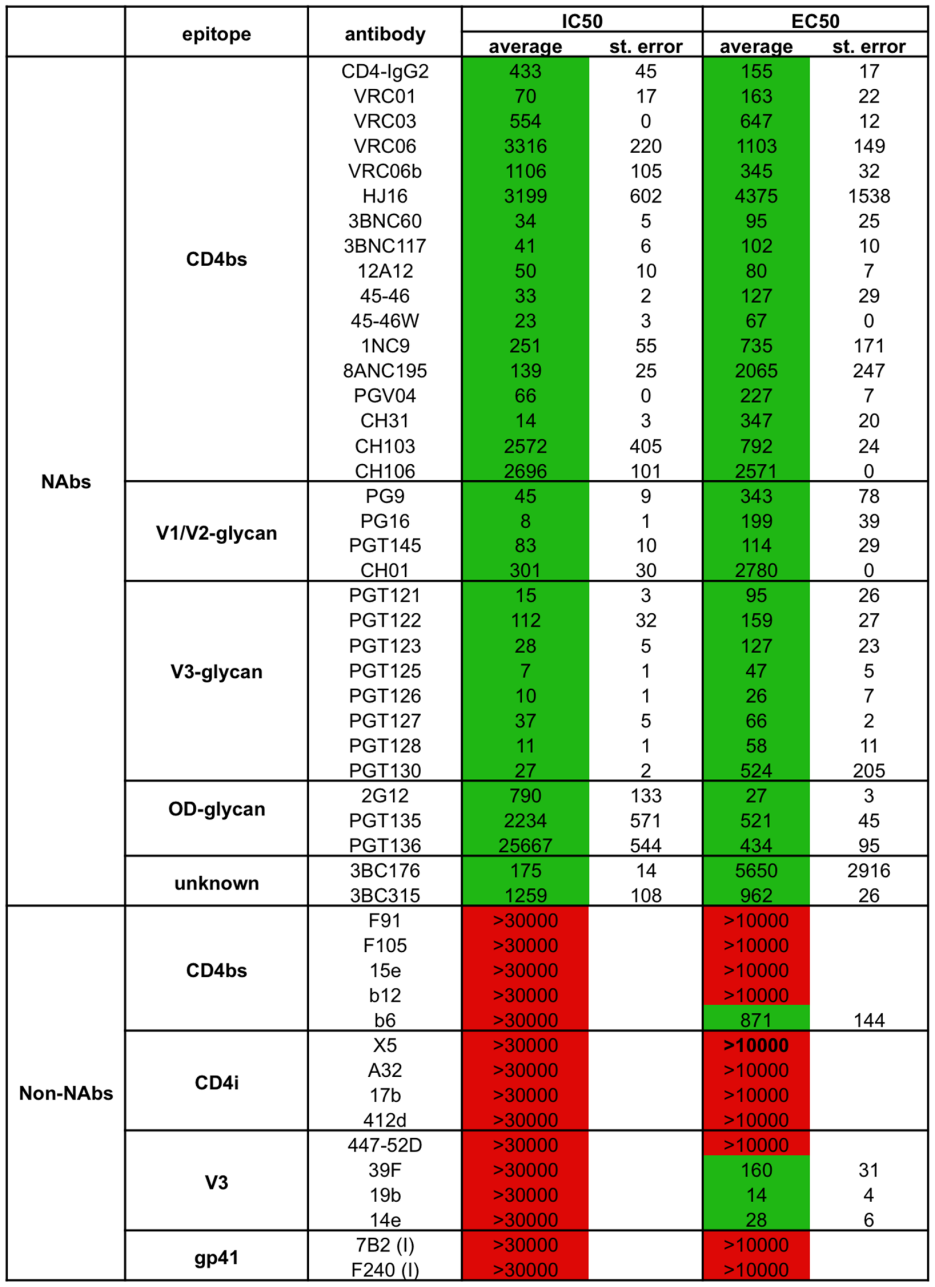
BG505.T332N virus neutralization and ELISA binding to BG505 SOSIP.664 gp140 trimers by bNAbs or non-NAbs. Midpoint neutralization concentrations (IC_50_, in ng/ml) were derived from single cycle experiments involving Env-pseudovirus infection of TZM-bl cells. The values represent the averages of 2–5 independent titration experiments, each performed in duplicate, with the standard error recorded. Half-maximal binding concentrations (EC_50_, in ng/ml) were derived from D7324-capture ELISAs. The values represent the averages of 2–6 independent single titration experiments, with the standard error recorded.

Most of the known bNAbs neutralized the BG505.T332N virus efficiently (Fig. 3). This outcome was true of bNAbs to the CD4bs (VRC01, VRC03, VRC06, VRC06b, PGV04, HJ16, 3BNC60, 3BNC117, 12A12, 45–46, 45–46W, 1NC9, 8ANC195, CH31, CH103, CH106 and also CD4-IgG2); the N332-glycan dependent V3 cluster (PGT121–123, PGT125–130); the N332-glycan dependent outer domain cluster (PGT135, PGT136, 2G12); 3BC315 and 3BC176; and the quaternary-dependent V1V2 epitopes (CH01, PG9, PG16, PGT145). Neutralization by PGT136 was modest (IC_50_, 26,600 ng/ml). Note that the CD4bs MAb b12 did not neutralize BG505 (IC_50_>30,000 ng/ml; Fig. 3), implying that it is a non-NAb for this subtype A virus [29]. The Duke University Central Laboratory has classified BG505.T332N as a Tier 2 virus, based on the use of Env-pseudotyped viruses in the TZM-bl cell assay.

Several test MAbs did not neutralize BG505.T332N (IC_50_>30,000 ng/ml), including b6, 15e, F91 and F105 to the CD4bs; 17b, 412d, X5 and A32 to CD4-induced epitopes; 447-52D, 39F, 19b and 14e to V3; F240 and 7B2 directed to gp41. We confirmed that their epitopes were present on at least one form of BG505 Env protein (e.g., gp120 monomers or gp41_ECTO_), showing that their inability to neutralize the virus was not due to a sequence-dependent lack of the epitope (data not shown, and see below). The CD4i MAbs 17b and 412d also did not neutralize the BG505.T332N virus when sCD4 was also present (data not shown). For the V3 MAbs 19b and 14e, we also performed extended pre-incubation experiments (16 h) before adding the MAb-virus mixtures to the target cells. Even in this assay format, the two V3 MAbs had no measurable neutralization activity, indicating that they do not inactivate the Env spike (data not shown).

### Antigenic analysis of BG505 SOSIP.664 gp140 trimers by ELISA

**Figure 4:**
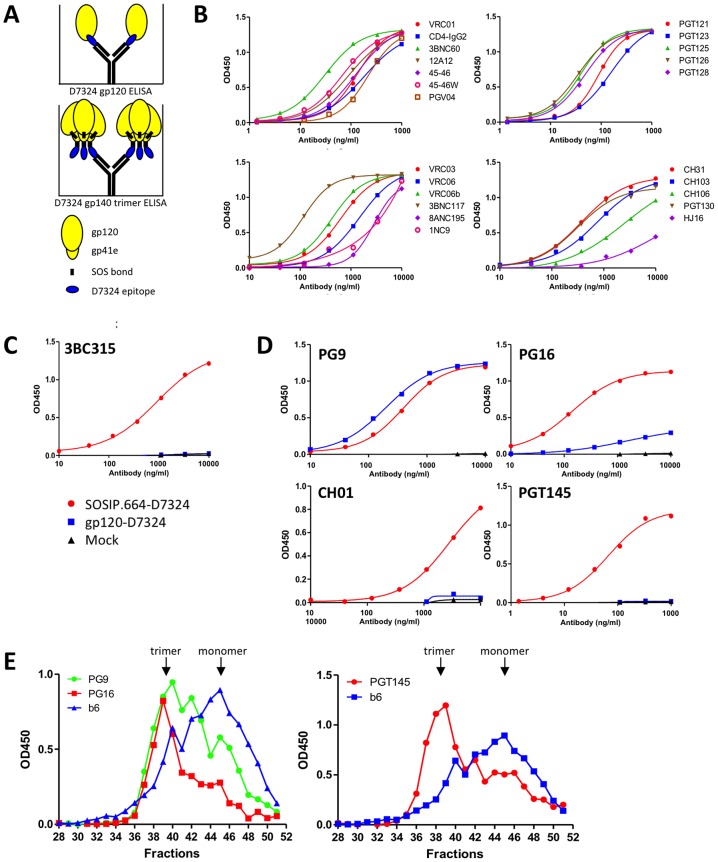
BG505 SOSIP.664 gp140 antigenicity by ELISA with bNAbs. (**A**) Schematic representation of D7324-capture ELISAs using BG505 gp120-D7324 monomers and/or SOSIP.664-D7324 gp140 trimers. (**B**) Representative binding curves of bNAbs VRC01, VRC03, VRC06, VRC06b, PGV04, 3BNC117, 12A12, 45–46, 45–46W, 1NC9, 8ANC195, CH31, CH103, CH106, PGT121, PGT123, PGT125, PGT126, PGT128, PGT130, and also CD4-IgG2, to purified BG505 SOSIP.664-D7324 gp140 trimers. (**C**) Representative binding curves of bNAb 3BC315 with purified BG505 SOSIP.664-D7324 gp140 trimers and gp120-D7324 monomers. (**D**) Representative binding curves of quaternary structure dependent bNAbs PG9, PG16, CH01 and PGT145 to purified BG505 SOSIP.664-D7324 gp140 trimers and gp120-D7324 monomers. The legend is the same as for panel C. (**E**) BG505 SOSIP.664-D7324 gp140 trimers were 2G12-affinity purified and fractionated using a Superose 6 10/30 SEC column. The SEC fractions were analyzed for PG9, PG16, PGT145 and b6 binding by D7324-capture ELISA. Note that the scales on the y-axes and x-axes vary from MAb to MAb.

**Figure 5:**
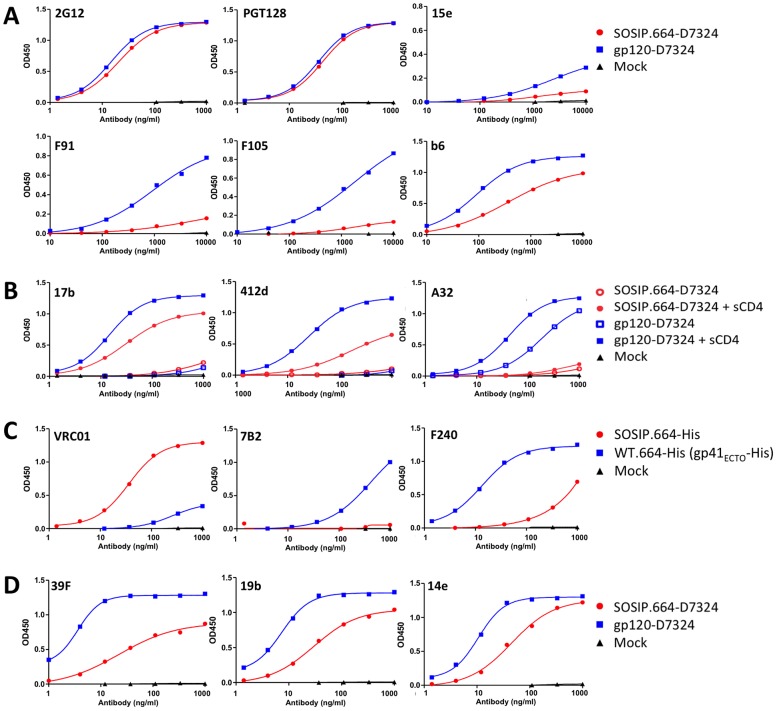
BG505 SOSIP.664 gp140 antigenicity by ELISA with non-bNAbs. Representative binding curves of: (**A**) gp120-directed non-NAbs 15e, F91, F105 and b6 to purified BG505 SOSIP.664-D7324 gp140 trimers and gp120-D7324 monomers, with bNAbs 2G12 and PGT128 included as controls. (**B**) CD4i MAbs 17b, A32 and 412d to purified BG505 SOSIP.664-D7324 gp140 trimers and gp120-D7324 monomers in the absence (open symbols) and presence (closed symbols) of sCD4. (**C**) gp41-directed non-NAbs 7B2 and F240 to BG505 SOSIP.664-His and WT.664-His ( = gp41_ECTO_-His) proteins. Both proteins were expressed in the presence of furin, yielding cleaved gp140. The lack of the SOS disulfide bond results in gp120 shedding from gp41_ECTO_-His, as illustrated by the poor reactivity with VRC01 (left panel). (**D**) V3 MAbs 39F, 19b and 14e to purified BG505 SOSIP.664-D7324 gp140 trimers and gp120-D7324 monomers. Note that the scales on the y-axes and x-axes vary from MAb to MAb.

We used several methods to quantify the binding of bNAbs and non-NAbs to wild type or epitope-tagged versions of BG505 SOSIP.664 gp140 trimers and, in some cases, the cognate SOSIP gp140 monomers, gp120 monomers, or gp41_ECTO_ proteins. First, we immobilized 2G12 affinity- and SEC-purified SOSIP.664-D7324 trimers onto ELISA plates and monitored the binding of a large panel of MAbs (Figs. 3–5). The immobilized trimers were recognized efficiently by all of the bNAbs against the CD4bs, the N332-glycan dependent V3 cluster or the N332-glycan dependent outer domain cluster that neutralized the corresponding virus (see above). The 3BC315 and 3BC176 bNAbs bind to an incompletely characterized, but probably glycan-independent, epitope that is induced, to an extent, by CD4 binding [38]. They do not bind to any soluble Env protein tested to date, including uncleaved soluble gp140 trimers [38]. However, both bNAbs interacted efficiently in ELISA with the BG505 SOSIP.664-D7324 gp140 trimers, but not the corresponding gp120 monomers (Fig. 3, Fig. 4C, and data not shown).

The bNAbs that recognize quaternary-preferring epitopes are particularly useful tools for gauging whether soluble Env trimers adopt an appropriate conformation. This bNAb category includes CH01, PG9, PG16 and PGT145 against epitopes that appear to span the V1V2 domains of two gp120s within a single trimer [27]. Although these bNAbs can bind a small subset of monomeric gp120s or uncleaved trimeric gp140s, any such interactions tend to be rare and weak, particularly for PG16 and PGT145 [12], [27], [29], [39]. Here, we show that CH01, PG9, PG16 and PGT145 all bound efficiently to the BG505 SOSIP.664 gp140 trimers in ELISA (Fig. 3, Fig. 4D). In contrast, only PG9 reacted well with monomeric BG505 gp120, while CH01 and PGT145 were completely non-reactive and PG16 bound weakly. We have also found that PG16 and PGT145 do not bind to BG505 SOSIP.664 gp140 trimer mutants that lack the glycans attached to N156 or N160, or to wild-type trimers produced in HEK293T cells treated with the mannosidase inhibitor kifunensine (data not shown). These findings are consistent with the known involvement of hybrid or complex glycans at N156 or N160 in the PG16 and PGT145 epitopes [30], [40].

To further study the influence of trimerization on the PG9, PG16 and PGT145 epitopes, we fractionated 2G12 affinity-purified SOSIP.664-D7324 gp140 proteins by SEC and analyzed the column fractions by ELISA (Fig. 4E). Both PG16 and PGT145 bound almost exclusively to the trimer-containing fractions, whereas PG9 bound more strongly to the trimers, but also recognized the SOSIP.664-D7324 gp140 monomers. This reactivity pattern is broadly consistent with previous observations on BG505 gp120 monomers [29]. In contrast, the CD4bs non-NAb b6 bound preferentially to the SOSIP.664-D7324 gp140 monomers, although some binding to trimers was also seen (see below).

For HIV-1 to be neutralized, a NAb must bind to a sufficient number of the native, functional, trimeric spikes present on the virus surface [41], [42]. Non-NAbs fail to neutralize because their epitopes are either absent from these trimers, or not accessible at the right time. A soluble gp140 trimer that mimics native, functional spikes should, therefore expose few or, ideally, no epitopes for non-NAbs. Accordingly, we assessed various non-NAbs for their abilities to bind wild type or epitope-tagged versions of BG505 SOSIP.664 gp140 trimers. Several non-NAbs to CD4bs epitopes (F91, F105, b6, 15e) did not bind the SOSIP.664-D7324 trimers or did so only weakly. However, all of them reacted strongly with the corresponding gp120 monomers and/or SOSIP.664 gp140 monomers (Fig. 3 and Fig. 5A). The diminished or absent reactivity of these non-NAbs with the trimers does not, therefore, reflect the absence of the epitope due to sequence variation, but rather the structural constraints present on “native-like” trimers.

We next investigated the binding of CD4i MAbs 17b, 412d, X5 and A32, which were all unable to neutralize the corresponding virus in the presence or absence of sCD4 (Fig. 3 and data not shown). None of these four non-NAbs bound to the BG505 SOSIP.664-D7324 gp140 trimers. When sCD4 was present, the 17b epitope was induced on the trimers (as were the similar 412d and X5 epitopes, to lesser extents), indicating that CD4-induced conformational changes had taken place (Fig. 3 and Fig. 5B, and data not shown). The 17b, 412d and X5 MAbs were also gp120 monomer-reactive but only when sCD4 was present (Fig. 3 and Fig. 5B, and data not shown). In contrast, the A32 MAb to a different category of CD4i epitope failed to bind the trimers even in the presence of sCD4, but bound strongly to the gp120 monomers in the absence of sCD4 and even more so when sCD4 was added (Fig. 3 and Fig. 5). The induction by sCD4 of conformational changes in the BG505 SOSIP.664-D7324 trimers, measured by ELISA, is consistent with the conformational changes seen in the EM images of sCD4/17b-complexes of the non-tagged trimers (see below).

Non-NAbs F240 and 7B2 against cluster I gp41_ECTO_ epitopes were minimally reactive with the SOSIP.664-D7324 or SOSIP.664-His gp140 trimers, but bound strongly to the corresponding WT.664-His (gp41_ECTO_) protein. Hence, these cluster I epitopes are present on BG505 gp41_ECTO_, but occluded by trimer formation (Fig. 3 and Fig. 5C).

In contrast to the above observations, the anti-V3 MAbs 39F, 19b and 14e, which did not neutralize the BG505.T332N virus, bound strongly to the BG505 SOSIP.664-D7324 gp140 trimers in ELISA, although less well than to the corresponding gp120 monomer (Fig. 3 and Fig. 5D). We note, however, that 19b and 14e bound only marginally to the same trimers in SPR- or EM-based assays (see below).

The following MAbs were also non-reactive for binding to BG505 SOSIP.664 gp140 trimers by ELISA: D50 (gp41 cluster II), 98-6 (gp41 cluster II), 48d (gp120 CD4i), 8K8 (gp41 HR1), DN9 (gp41 HR1), CH58 (gp120 V2), CH59 (gp120 V2), HG107 (gp120 V2), HG120 (gp120 V2). However, we could obtain no evidence for their reactivity with any other form of BG505 Env protein; i.e., gp120 monomer, SOSIP gp140 monomer or gp41_ECTO_-His (data not shown). We therefore conclude that the epitopes for these MAbs are absent from BG505 Env proteins due to sequence variation, negating their value for assessing trimer antigenicity.

**Figure 6:**
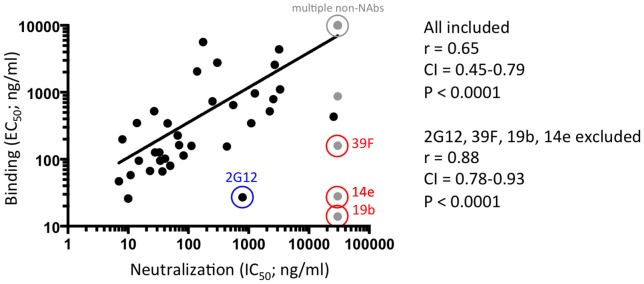
Correlation between MAb binding to BG505 SOSIP.664 gp140 trimers and BG505.T332N neutralization. The midpoint binding concentrations (EC_50_) for MAb binding to BG505 SOSIP.664-D7324 gp140 trimers (y-axis) were plotted against the IC_50_ values for neutralization of the BG505.T332N Env-pseudotype virus (x-axis). The Pearson's correlation coefficient, r, was calculated using Prism software version 5.0. When accurate midpoint concentrations could not be calculated because of lack of binding or neutralization, the highest concentration tested was included in the correlation analysis (i.e., when the IC_50_ of neutralization was >30,000 ng/ml, a value of 30,000 ng/ml was used). The data points for NAbs are indicated in black, those for non-NAbs in gray. Also note that 11 data points are overlapping in the right upper corner; they were derived using MAbs that neither neutralized the virus nor bound the trimer (IC_50_ of neutralization >30,000 ng/ml, EC_50_ in ELISA >10,000 ng/ml). Only MAbs whose epitope could be shown to be present on at least one form of BG505 Env protein were included in this analysis; MAbs that were non-reactive, presumably because of sequence variation, were excluded. The Pearson's correlation was also calculated without the data points for 2G12, 39F, 14e and 19b, as discussed in the text. The fitted line is based on all data, i.e. including 2G12, 39F, 14e and 19b.

We plotted the EC_50_ values for MAbs to the SOSIP.664-D7324 gp140 trimers against the IC_50_ values for neutralization of the corresponding BG505.T332N Env-pseudovirus (Fig. 6). The resulting Spearman's correlation coefficient, r, was 0.65 (95% confidence interval 0.45–0.79), which was highly significant (P<0.0001). Thus, the BG505 SOSIP.664 gp140 trimer is an excellent antigenic mimic of the functional native BG505.T332N Env spike.

Most non-NAbs did not bind the SOSIP.664-D7324 gp140 trimers in ELISAs, or did so only weakly compared to their reactivity with monomeric proteins. The most striking outliers were the V3 non-NAbs 39F, 14e and 19b, which did bind strongly in this assay (Fig. 5D). The bNAb 2G12 bound more strongly to trimers in ELISA than would be predicted by its neutralization capacity. This outcome might be attributable to the specific enrichment of 2G12-reactive soluble trimers during the affinity purification process, given that other, less 2G12-reactive Env spikes will contribute to infection during neutralization assays. When 2G12, 39F, 14e and 19b were excluded from the correlation, the r-value increased to 0.88 (95% confidence interval 0.80–0.94; P<0.0001).

### Antigenic analysis of BG505 SOSIP.664 gp140 trimers by surface plasmon resonance

**Figure 7:**
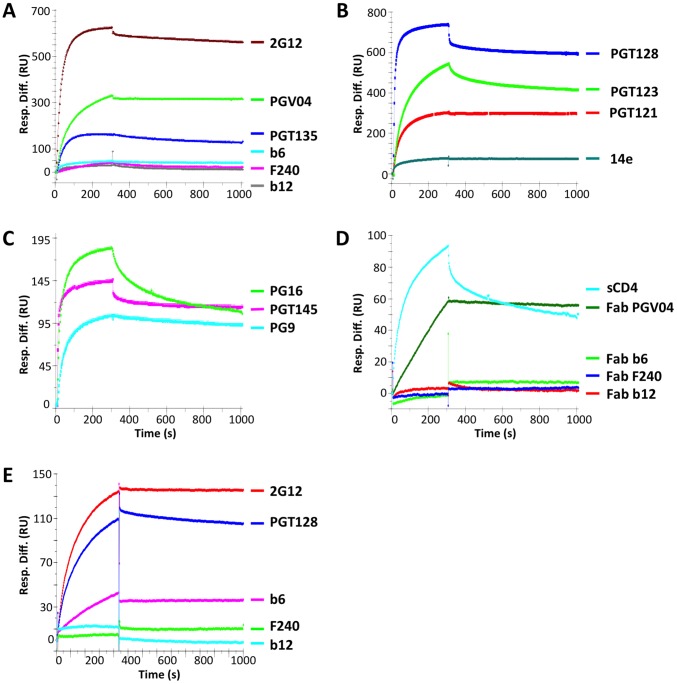
BG505 SOSIP.664 gp140 antigenicity by SPR. BG505 SOSIP.664-His gp140 trimers were immobilized on NTA chips (**A**–**D**). The sensorgrams show the response (RU) over time (s) using IgGs at 1,000 nM (150,000 ng/ml) (A–C) or Fabs at 500 nM (25,000 ng/ml) (D). The association phase was 300 s and dissociation was followed over 600 s. (**A**) 2G12 (high), PGT135 (intermediate), PGV04 (high), b6 (marginal), b12 (undetectable) and F240 (undetectable); (**B**) PG9, PG16, PGT145 (all intermediate); (**C**) PGT121 (intermediate), PGT123 (high), PGT128 (high), 14e (low); (**D**) Fabs of PGV04 (intermediate), b6, b12, and F240 (undetectable). (**E**) In an alternative approach, Env-reactive MAb was captured by anti-Fc Ab on the chip and the responses to BG505 SOSIP.664 gp140 trimers (200 nM; 78,000 ng/ml) were followed: 2G12 (high), PGT128 (high), b6 (low), b12, F240 (undetectable). Each curve represents one of 2–3 similar replicates.

We next investigated the binding of a subset of representative bNAbs and non-NAbs using surface plasmon resonance (SPR). In this assay, binding of MAbs to BG505 SOSIP.664-His gp140 trimers, immobilized via His-Ni^2+^ interaction on NTA chips, again generally agreed well with their capacity to neutralize the corresponding Env-pseudovirus (Fig. 7A–C). Thus, bNAbs 2G12 and PGT135 to glycan-dependent epitopes on the outer domain of gp120, bound to high and intermediate levels, respectively, in the SPR assay. The PGV04 bNAb (CD4bs) bound strongly with markedly slow dissociation, the V1V2- and quaternary-structure-dependent bNAbs PG9, PG16, and PGT145 all bound to intermediate levels, while the V3- and N332-dependent bNAbs PGT121, PGT123, and PGT128 bound to intermediate or high extents with distinctive kinetics. In contrast, the CD4bs non-NAb b6 reacted only marginally with the trimers in the SPR assay, while b12 (CD4bs) and F240 (gp41 cluster I) did not bind detectably. The V3-specific non-NAb 14e, which did bind strongly to BG505 SOSIP.664-D7324 gp140 trimers in ELISA, was only marginally reactive with the corresponding His-tagged trimers by SPR; the low signals for 14e contrast markedly with those for the V3- and N332-dependent bNAbs (e.g., the plateau values were 60–70 RU and 750 RU for 14e and PGT128, respectively). Moreover, the plateau signal for 14e at 1,000 nM (150,000 ng/ml) was only twice that for b6 (30 RU).

An even starker contrast between effective binding and complete lack of interaction was observed when Fabs were used instead of IgG molecules: the PGV04 Fab (bNAb) bound to an intermediate level (when the three times lower mass contribution to the resonance is taken into account), whereas there was no detectable binding of the (non-NAb) Fabs b6, b12 or F240 (Fig. 7D). Soluble CD4 (of a similar mass to Fabs) bound to a somewhat higher level than the PGV04 Fab, but with markedly faster association and dissociation kinetics (Fig. 7D).

The converse SPR approach of immobilizing the Env-reactive Abs and allowing the untagged BG505 SOSIP.664 gp140 trimers (at 200 nM; 78,000 ng/ml) to bind from the solution phase yielded broadly similar results for the subset of MAbs tested in this way (Fig. 7E). Thus, strong responses were obtained for trimer binding to immobilized 2G12 or PGT128. The BG505 SOSIP.664 trimers did not bind detectably to the immobilized b12 or F240 IgG (non-NAbs) in this SPR format, but a low level of binding to the b6 IgG (also a non-NAb) was observed. The extent of trimer-b6 binding was greater than in the converse SPR set-up, perhaps because the intrinsically weak paratope-gp120 binding is compensated for by the avidity effect of potentially trivalent interactions with the captured IgG; the 2.7-fold larger mass of the trimer compared to IgG should also be taken into account when assessing the degree of binding.

### Antigenic analysis of BG505 SOSIP.664 gp140 trimers by isothermal titration calorimetry

**Figure 8:**
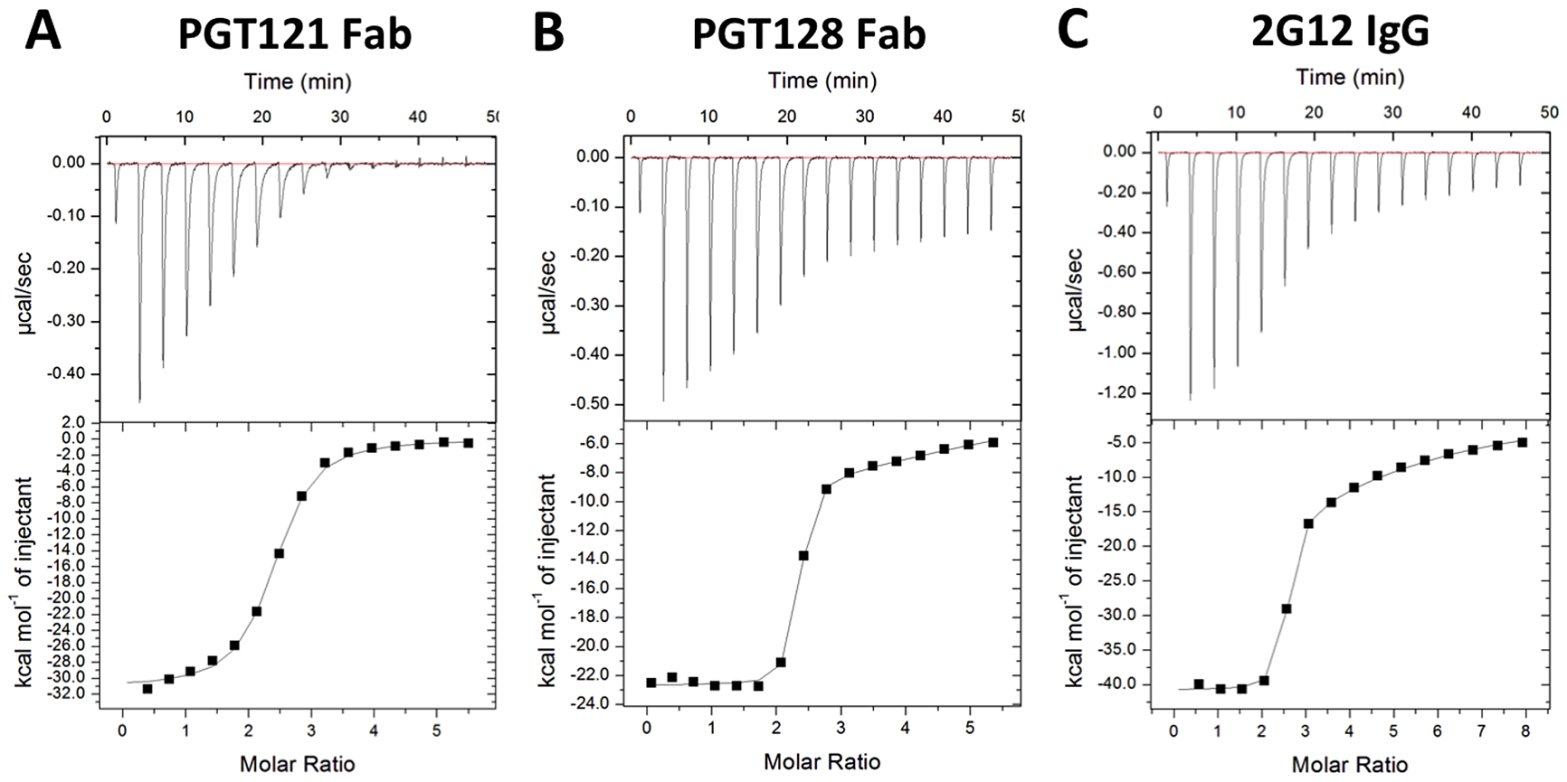
BG505 SOSIP.664 gp140 trimer antigenicity by ITC. The top panels show the raw data and the bottom panel the binding isotherms for representative ITC binding experiments measuring the binding of BG505 SOSIP.664 gp140 trimers to: (**A**) PGT121 Fab, (**B**) PGT128 Fab, (**C**) 2G12 IgG (domain-exchanged). The thermodynamic parameters of binding are listed in Table 1.

To determine the thermodynamic binding characteristics of the BG505 SOSIP.664 gp140 trimers, we performed isothermal titration calorimetry (ITC) experiments using PGT121 Fab, PGT128 Fab and the domain-exchanged 2G12 IgG. All three antibodies have previously been shown to be dependent on the high-mannose glycan at position N332 for Env recognition [30], [43], [44], [45], [46]. PGT121, PGT128 and 2G12 all bound the BG505 SOSIP.664 trimer with nanomolar affinities (151 nM = 7550 ng/ml; 5.7 nM = 284 ng/ml; and 16.0 nM = 2400 ng/ml, respectively) and near identical stoichiometries of 2.3–2.4 (Table 1; Fig. 8). These binding stoichiometries are three-fold higher than the value of 0.8 previously reported for PG9 binding (affinity 11 nM = 550 ng/ml) to the same construct (Table 1) [27]. The data therefore imply that three PGT121 Fabs, PGT128 Fabs or 2G12 IgG molecules bind per trimer, which is consistent with their recognition of N332-dependent epitopes on the outer domain of gp120. In contrast, only a single PG9 Fab recognizes the N160-dependent epitope at the membrane-distal apex of each trimer [27]. Taken together, the ITC binding data further confirm that the BG505 SOSIP.664 gp140 trimers properly display the high-affinity binding sites for the glycan-dependent bNAbs, PGT121, PGT128, 2G12 and PG9.

**Table 1:** Thermodynamic parameters of PGT121, PGT128, 2G12 and PG9 binding to BG505 SOSIP.664 gp140 trimers measured by isothermal titration calorimetry.

Binding experiment	ΔG^a^^,^ b (kcal mol^−1^)	ΔH^a^ (kcal mol^−1^)	-TΔS^a^ (kcal mol^−1^)	K_d_ a (nM)	K_d_ a (ng/ml)	N^a^^,^ c	K_d2_ a ^,^ d (nM)
PGT121 Fab into BG505 SOSIP.664	−9.3	−29.9	20.6	151	7550	2.4	N/A
PGT128 Fab into BG505 SOSIP.664	−11.2	−22.8	11.6	5.7	285	2.3	20,000^e^
2G12 IgG into BG505 SOSIP.664	−10.6	−39.0	28.4	16.0	2400	2.4	12,300^e^
PG9 Fab into BG505 SOSIP.664^f^	−10.9	−18.7	7.8	11.0	550	0.8	N/A

a The reported values are averages from at least two independent measurements. The associated errors are approximately 10% of the average. Representative isotherms are shown in Fig. 8.

b The change in Gibbs free energy (ΔG) was determined using the relationship: ΔG_binding_ = RTlnK_d_ [87].

c The stoichiometry of binding (N) is directly affected by errors in protein concentration measurements, sample impurity and heterogeneity of gp140 glycans.

d Dissociation constant associated with a second (low affinity) binding event.

e The binding isotherms do not allow the stoichiometry and enthalpy associated with the second binding event to be determined accurately.

f Data previously described elsewhere [27].

### Antigenic analysis of BG505 SOSIP.664 gp140 trimers by electron microscopy

**Figure 9:**
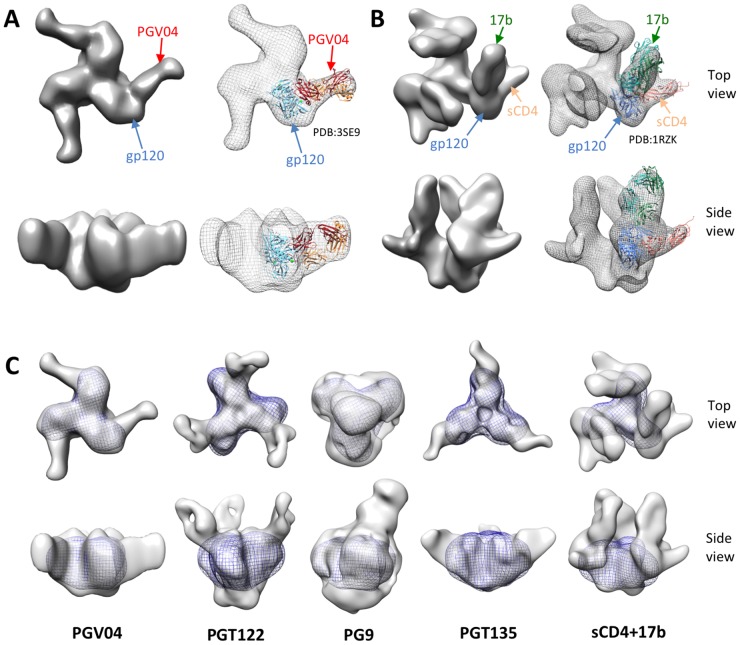
BG505 SOSIP.664 gp140 trimer antigenicity by negative stain EM. (**A**) EM reconstruction of the BG505 SOSIP.664 gp140 trimer in complex with Fab PGV04 at 23 Å resolution. The 2D class averages and the Fourier Shell Correlation (FSC) are shown in Fig. S3. The crystal structure of gp120 in complex with PGV04 (PDB:3SE9; [85]) was fitted into the EM density. (**B**) EM reconstruction of the BG505 SOSIP.664 gp140 trimer in complex with sCD4 and Fab 17b at 22 Å resolution. The 2D class averages and the Fourier Shell Correlation (FSC) are shown in Fig. S4. The crystal structure of gp120 in complex with sCD4 and 17b (PDB:1RZK; [86]) was fitted into the EM density. (**C**) Comparison of unliganded BG505 SOSIP.664 gp140 trimers with complexes of the same trimers with PGV04, PGT122, PG9, PGT135, or sCD4 with 17b. The unliganded trimer is shown in mesh. The EM reconstructions of PGT122, PG9, PGT135 with BG505 SOSIP.664 trimers, expressed in HEK293S cells, have been published elsewhere [25], [26], [27].

We used negative stain electron microscopy (EM) to characterize the binding of the CD4bs bNAb PGV04 to the BG505 SOSIP.664 gp140 trimer (Fig. 9A; Fig. S3). The reconstruction at 23-Å resolution shows that PGV04 binds the soluble trimers in a manner similar to other CD4bs-directed bNAbs with virion-associated Env, in that it approaches the gp120 protomers from the side [47]. We compared the complex formed between PGV04 and BG505 SOSIP.664 trimers with other such bNAb-trimer complexes. Recent studies with the same trimers, albeit expressed in glycan processing-deficient GnTI^−/−^ HEK293S cells and not, as here, HEK293T, have shown how the PGT122 and PGT135 bNAbs bind to their N332 glycan-dependent epitopes. Thus, their angle of approach differs from how PGV04 encounters the CD4bs, but all three bNAbs saturate the three available binding sites on the trimer (Fig. 9C) [25], [26]. In contrast, and as noted above, the quaternary preferring, N160 glycan-specific bNAb PG9, only binds to one epitope per trimer (Fig. 9C) [27]. Images of the BG505 SOSIP.664 trimers in complex with sCD4 and 17b show that conformational changes are induced (Fig. 9B) that are consistent with ones described for SOSIP trimers based on the JR-FL and KNH1144 genotypes [23], [24], [48], and for the full length, virus-associated BaL Env spike [49]. Collectively, the new and recently published negative stain EM data show that BG505 SOSIP.664 gp140 trimers, derived from HEK293T or HEK293S cells, express multiple different bNAb epitope clusters, and also undergo conformational changes when they bind sCD4 [25], [26], [27].

**Figure 10:**
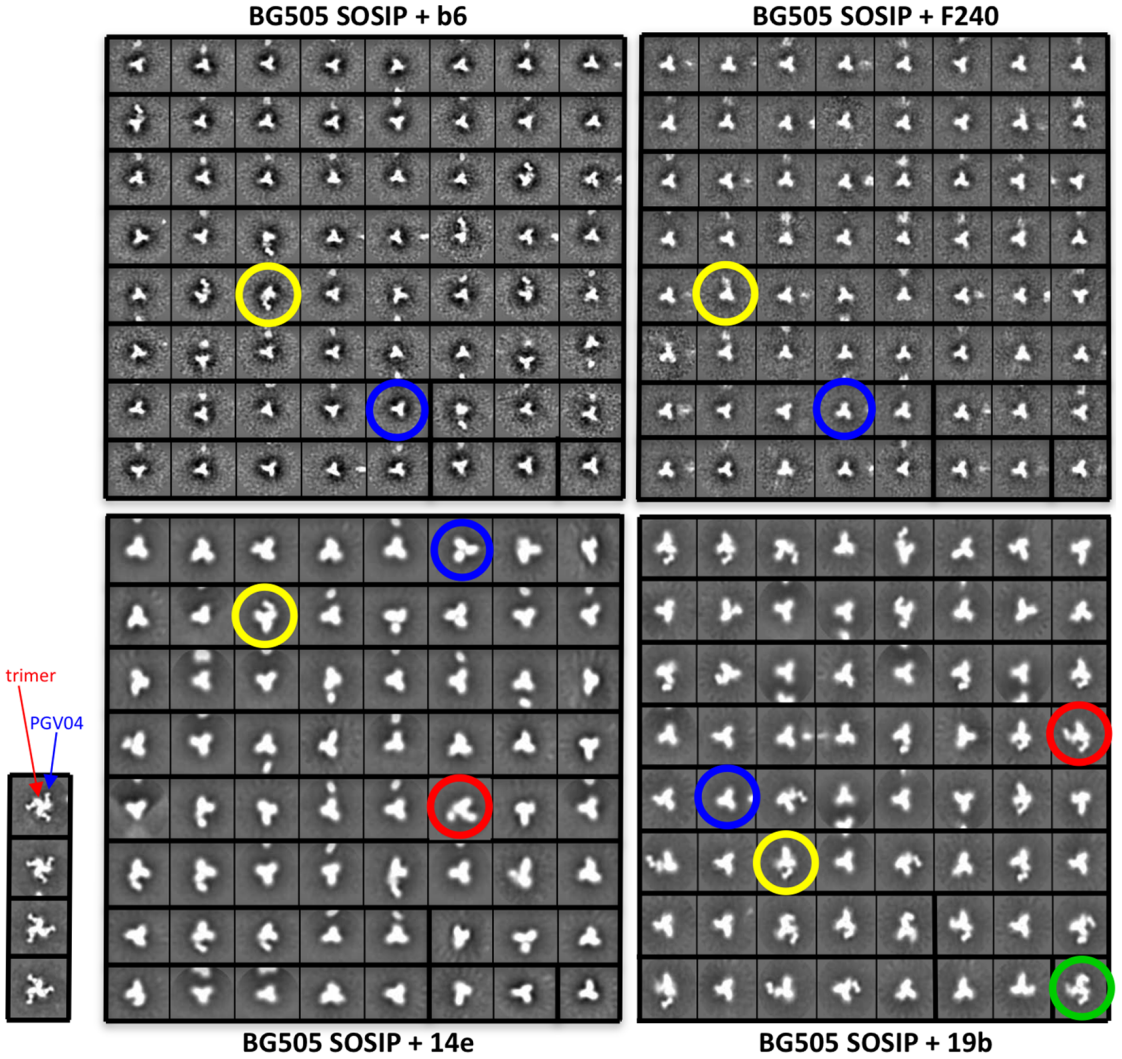
Negative stain EM data of the BG505 SOSIP.664 gp140 trimer in complex with Fabs b6, F240, 14e or 19b. 2D class averages of trimers and trimer∶Fab complexes, with 2D class averages of BG505 SOSIP.664 gp140 with PGV04 shown on the lower left panel for comparison. Examples of complexes are circled. Blue circle: no Fab bound to trimer; yellow circle: one Fab bound; red circle: two Fabs bound; green circle: three Fabs bound. For generating b6 complexes, BG505 SOSIP.664 (44 nM; 17,200 ng/ml) was incubated with Fab b6 (480 nM; 24,000 ng/ml) prior to imaging. For generating F240 complexes, BG505 SOSIP.664 (48 nM; 18,720 ng/ml) was incubated with Fab F240 (860 nM; 43,000 ng/ml) prior to imaging. For generating 14e complexes, BG505 SOSIP.664 (14 nM; 5460 ng/ml) was incubated with Fab 14e (240 nM; 12,000 ng/ml). For generating 19b images, BG505 SOSIP.664 (40 nM; 15,600 ng/ml) was incubated with Fab 19b (600 nM; 30,000 ng/ml). The results are summarized in Table 2.

We also collected images of mixtures of the BG505 SOSIP.664 gp140 trimers with non-NAbs b6, 14e, 19b and F240, added as Fabs and in molar excess (Table 2; Fig. 10). Essentially none (<3%) of the trimers bound to F240, while ∼6% could be seen to have a single b6 Fab attached (Table 2; Fig. 10). Hence the EM images are concordant with the ELISA and SPR data for F240. The ELISA-reactive V3 non-NAbs 14e and 19b bound the trimers to only a limited extent by EM (Table 2; Fig. S4, S5). Thus, ∼19% of the trimers were occupied by one (∼17%) or two (∼2%) 14e Fabs, and ∼39% by one (∼30%), two (∼8%) or three (∼1%) 19b Fabs.

**Table 2:** Binding of non-NAbs to BG505 SOSIP.664 gp140 trimers as observed by negative stain EM.^a^

Fab	Total	Trimer alone		1 Fab bound		2 Fabs bound		3 Fabs bound	
	particles	particles	%	particles	%	particles	%	particles	%
b6	5964	5610	94	354	6	0	0	0	0
14e	26908	21854	81	4444	17	610	2	0	0
19b	40833	25111	61	12457	30	3054	8	211	1
F240	6417	6263	98	154	2	0	0	0	0

a Representative class averages are shown in Fig. 10.

Overall, the observations made with the non-NAbs b6, 14e, 19b and F240 contrast markedly with the EM images of the same trimers in complexes with bNAbs PGT122, PGT135 and PGV04, where there was full occupancy (i.e., three Fabs bound) in >50% of the images and no occupancy (i.e., no Fabs bound) in only <3% of the images (not shown).

### Antigenic analysis of BG505 SOSIP.664 gp140 trimers: Summary

**Figure 11:**
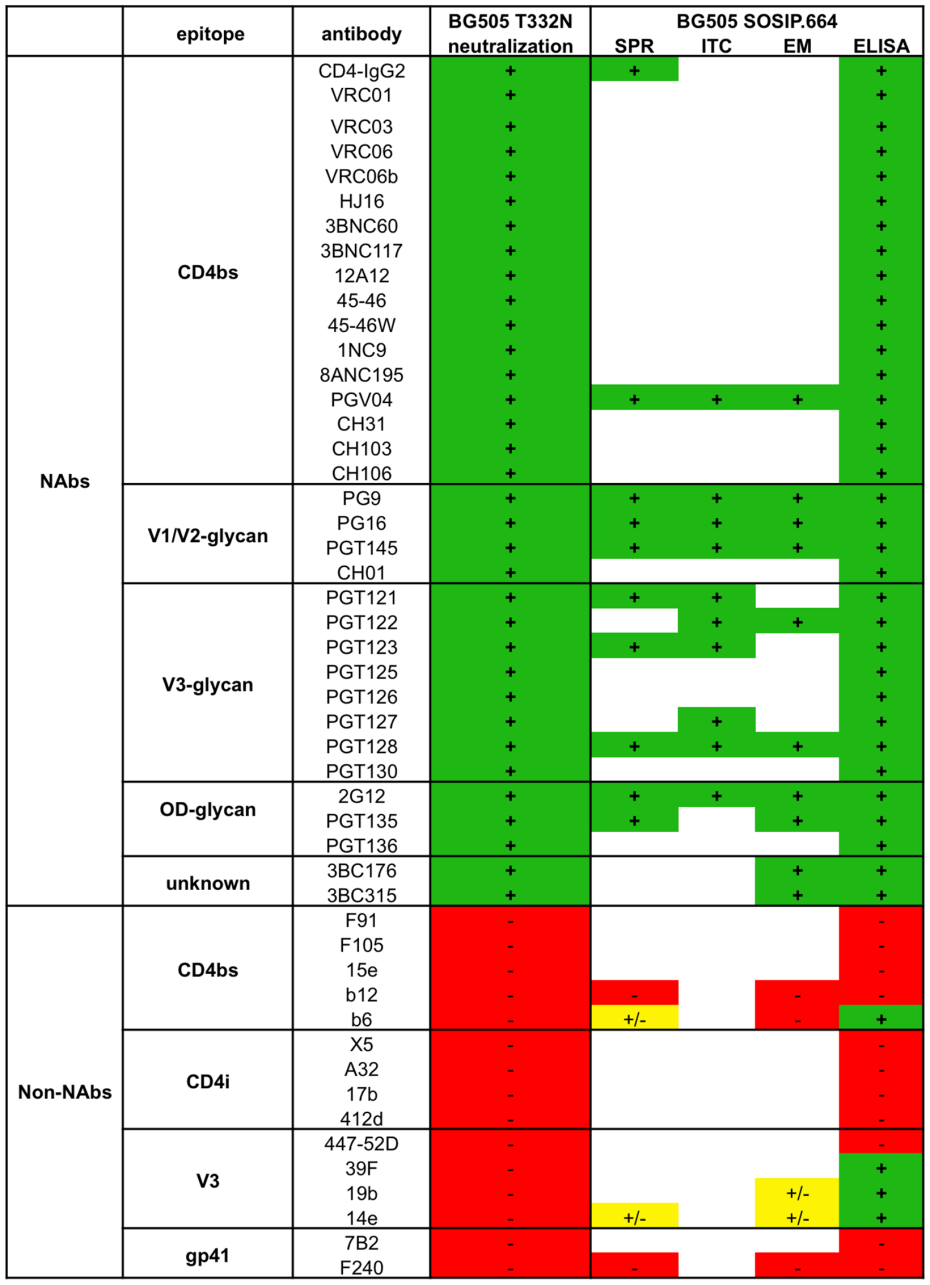
BG505 SOSIP.664 antigenicity summary for bNAbs and non-NAbs. The following scoring was used for neutralization: +: IC_50_<30,000 ng/ml; −: IC_50_>30,000 ng/ml. See Table 1 for details. The following scoring was used for SPR analyses: +: >70 RU; +/−: >30 RU, <70 RU; −: <30 RU, based on plateau estimates of 1,000 nM for IgG and 500 nM for Fab. See Fig. 7 for details. In SPR CD4 binding results were obtained with sCD4 not CD4-IgG2. b6 binding was absent with b6 Fab (−) and low with b6 IgG (+/−). The following scoring was used for ITC experiments: +: N>1.2 (except for PG9, PG16 and PGT145 where N>0.4) and K_d_<300 nM; −: N<1.2 and K_d_>300 nM, where N is the stoichiometry of binding. See Table 1 and Fig. 8 for details. . The following scoring was used for EM analyses: +: N = 3 (except for PG9, PG16 and PGT145 where N = 1) for >50% of the trimers; +/−: N = 3 for <50% of the trimers; −: N = 1,2 or 3 for <10% of the trimers, where N is the number of Fabs bound per trimer. See Table 2, Fig. 9 and Fig. 10 for details. Note that ITC data with PG16 and PGT127 were obtained using BG505 SOSIP.664 trimers produced in GnTI-defective HEK293S cells. No ITC experiments were performed with non-NAbs. The following scoring was used for ELISA experiments: +: EC_50_<10,000 ng/ml; EC_50_>10,000 ng/ml. See Figs. 3–5 for details.

Taken together, the various antigenicity assays show that every MAb that neutralizes the BG505.T332N virus efficiently also bound strongly to the soluble BG505 SOSIP.664 gp140 trimers (wild type and/or epitope tagged), except for MAbs to MPER epitopes that were not present in the trimer construct (data not shown) (Fig. 11). We conclude that the trimers display a range of bNAb epitopes from multiple different clusters. In contrast, non-NAb epitopes are generally structurally occluded and not displayed on the SOSIP trimer (Fig. 11).

## Discussion

We describe here the design and properties of a next-generation, fully cleaved and highly stable soluble gp140 trimer based on the BG505 subtype A sequence. EM imaging shows that the BG505 SOSIP.664 gp140 trimers are homogeneous and that their architecture is very similar to that of native Env spikes on virions. We used a range of techniques to assess the antigenic properties of the soluble trimers, particularly their abilities to bind bNAbs and non-NAbs and the relationship between trimer binding and virus neutralization. The various techniques were generally concordant, with one notable exception relating to V3 MAbs. Overall, there were few discrepancies between the antigenicity of the trimers and the neutralization sensitivity of the corresponding BG505.T332N virus. Thus, in general, all the bNAbs that neutralized the virus also bound to the soluble trimers, with the obvious exception of bNAbs to the MPER, a region that was eliminated from the trimers to improve their biophysical properties. The presence of so many bNAb epitopes on a soluble, generally homogenous and highly stable trimer is highly beneficial for structural studies, and may also be valuable for their immunogenicity properties. Whether the favorable antigenic profile translates into the induction of bNAbs will be determined experimentally.

In contrast to bNAbs, non-NAbs were rarely strongly reactive with the BG505 SOSIP.664 gp140 trimers, even when their epitopes were present on less complex forms of BG505 Env (e.g., gp120 or gp140 monomers, or gp41_ECTO_). This finding was particularly striking for non-NAbs against the CD4bs, such as F91 and F105, and implies that the steric constraints on MAb access to this region of virion-associated Env also applies to the soluble trimers. The same argument applies to the various CD4i epitopes, which were inaccessible on the soluble trimers unless sCD4 was also bound, and to non-NAb epitopes in gp41_ECTO_. We note that the A32 epitope, while present and further induced by sCD4 on BG505 gp120 monomers, was absent from the trimers whether sCD4 was present or not. This observation may be relevant to arguments that the A32 epitope is an important target for ADCC-mediated killing of infected cells [50]. The only discordance between the trimer-binding and virus-neutralization assays involved the V3 region of gp120. Thus, the V3 non-NAbs 19b, 14e and 39F bound efficiently to the D7324-tagged trimers in the capture ELISA. However, the outcomes of the SPR and negative stain EM assays were quite different, in that, in these assays, the V3 MAbs were only minimally reactive with His-tagged, D7324-tagged or non-tagged trimers. One explanation may be that the capture onto the ELISA plate via the D7324 antibody might induce some local unfolding of the trimers. As we think it unlikely that the low level of binding of 19b and 14e seen by EM can explain the rather strong binding in ELISA, we favor this explanation but acknowledge that it is speculative in nature. We do, however, note that, at high concentrations, some binding of a subset of other non-NAbs can be observed in ELISA (Fig. 5). It is therefore also possible there is some conformational heterogeneity in the trimer population, with a minor subset displaying some non-NAb epitopes. Negative stain EM does show that some trimers can bind one or (very rarely) two non-NAb Fabs (Fig. 10). Another possibility is that the trimers are flexible, allowing different conformations to be sampled over time in a way that registers more strongly in an ELISA than in other binding assays [51], [52].

We note that an absolute stoichiometry of <3 (i.e., 2.4) was found for 2G12 IgG in the ITC binding experiments, whereas a value of 3 might have been expected for trimers that had been affinity-purified on a 2G12-IgG column. The discrepancy might arise from errors in glycoprotein concentration measurements. Sample impurity can also contribute to lower apparent binding stoichiometries. However, another possibility is that some gp120 protomers on a trimer do not express the 2G12 epitope due to variation in the glycosylation process. The presence of one or two 2G12 epitopes per trimer is probably sufficient for binding to the 2G12 affinity column. Of note is that ITC also yielded trimer-binding stoichiometries of 2.3 to 2.4 for the glycan-dependent Fabs PGT121 and PGT128, whereas Fab PGT122, which is very similar to PGT121, saturated all three binding sites on the trimer as visualized by EM (Fig. 9B). The explanation(s) might be similar to those suggested for 2G12.

In the ITC experiments, a weak secondary binding event could be seen for the PGT128 and 2G12 bNAbs, in addition to the saturating high-affinity event. PGT128 and 2G12 have high affinities for mimetic (i.e., non-Env) oligomannose glycan substrates, and might therefore interact weakly with other oligomannose glycans on the SOSIP.664 gp140 trimers, in addition to their high-affinity epitopes. Whether such low affinity binding events imply that high concentrations of these antibodies would react with secondary binding sites on the virion-associated Env trimer, and hence contribute to neutralization, is not yet known.

Across the entire bNAb and non-NAb test panel, there was an excellent concordance between the outcomes of trimer-reactivity (by ELISA) and virus-neutralization assays. Thus, a formal correlation plot yielded a highly significant r-value of 0.65 (P<0.0001). Such an outcome would not be the case for Env-binding assays using gp120 monomers or uncleaved gp140 trimers, because of their strong reactivity with multiple non-NAbs [5], [20], [21], [22]. The V3 non-NAbs were the predominant outliers in the correlation analysis and, as noted above, their strong ELISA reactivity is not supported by the SPR and EM studies. One other outlier was the 2G12 bNAb. Here, the discrepancy is likely to be rooted in the use of a 2G12-affinity column for purifying the BG505 SOSIP.664 gp140 trimers (D7324-tagged or not). Thus, the column is likely to select for a trimer sub-population that has a high affinity for 2G12. In contrast, the virions used in neutralization assays undergo no such 2G12-selection procedure and would have a more “average” affinity for this bNAb. When 2G12 and the V3 MAbs were excluded, the r-value for the trimer-reactivity and neutralization correlation increased to 0.88 (P<0.0001). Overall, we are encouraged by the general occlusion and/or absence of non-NAb epitopes on the BG505 SOSIP.664 gp140 trimers, not only because of their antigenic, and arguably structural, fidelity with respect to virion-associated trimers, but also for immunogenicity studies. Thus, when the goal is to induce bNAbs, non-NAb epitopes represent a distraction to the immune system that is best avoided. Less sophisticated Env immunogens that do not mimic the native spike efficiently induce non-NAbs, if and when this is a desired outcome [3], [53]. It is possible that the exposure of some V3-associated non-NAb epitopes on the BG505 SOSIP.664 trimers under certain experimental conditions *in vitro* (e.g., in the ELISA) might also occur when they are used as immunogens. While V3 is not an important neutralization site for primary viruses, some V3 Abs are active against a subset of viruses *in vitro*. Hence, any induction of V3 Abs in *vivo* might be useful under some circumstances. A converse argument, however, is that V3 is an immunodominant epitope cluster that may distract the immune system from focusing on more worthwhile targets elsewhere on the trimer. If so, it would be best to mask or stabilize the V3 region on a new variant of BG505 SOSIP.664 gp140 trimers, for example by introducing a glycan site(s) at an appropriate position [54]. Care would need to be taken, however, to occlude only undesirable regions of V3 (e.g., the 19b/14e sites) without affecting nearby areas that contribute to genuine bNAb epitopes, such as those for PGT121–123 and PGT125–130. We also note that glycan-masking might introduce unwanted neo-epitopes. Additional structural information that would help guide future SOSIP gp140 trimer re-designs is being actively sought.

It may never be possible to make a soluble gp140 trimer that precisely mimics the native Env spike, because deleting the transmembrane and cytoplasmic domains (and, in the case of SOSIP.664 trimers, also the MPER) will have at least some impact on trimer structure. Thus, point substitutions in gp41 HR1, HR2, MPER and the intracytoplasmic tail, as well as the length of the cytoplasmic tail, can influence the interaction of antibodies with the gp120 moieties of trimers on virions and infected or transfected cells [55], [56], [57], [58], [59]. Nonetheless, the BG505 SOSIP.664 gp140 trimers, as assessed by a variety of different antigenicity assays, do seem to come very close to being a faithful Env-spike mimetic. Ongoing structural studies will help confirm or refute this assessment. Whether the properties of the present trimers can be further improved by a targeted mutagenesis approach remains to be determined. We will show elsewhere just how critical cleavage of the gp120-gp41 linkage is for making soluble trimers that resemble native spikes.

The BG505 SOSIP.664 gp140 trimers have already been already useful for structure-based studies aimed at defining bNAb-Env interactions [25], [26], [27], as were the corresponding trimers based on the KNH1144 *env* gene [46], [48], [60]. The BG505 trimers are also substrates for ongoing efforts aimed at defining crystal or high-resolution EM structures of the Env trimer. In addition, we are assessing their immunogenicity in rabbits, guinea pigs and macaques, as well as determining how to make them in the much larger quantities, and of the appropriate quality, required for any future testing in humans. A high priority will also be to identify additional genotypes that yield SOSIP.664 trimers with the same favorable properties as the ones described here. Among hypotheses to explore is that the T/F and/or pediatric status of the BG505 isolate is relevant to the trimers' homogeneity and stability; a second relates to the presence of certain trimer-stabilizing residues associated with thermal stability. Thus, the M535, N543 and K567 residues that are present in BG505 SOSIP.664 have been reported to contribute to the trimerization efficiency of soluble gp140 and the thermal stability of Env trimers on virus particles [61], [62].

## Methods

### Construct design

The BG505 (BG505.W6M.ENV.C2) *env* gene (GenBank accession nos. ABA61516 and DQ208458) is derived from a subtype A T/F virus isolated from a 6-week old, HIV-1-infected infant [28]. It has 73% identity to the proposed PG9-sensitive progenitor virus from the PG9 bNAb donor, based on computational analysis of the most recent common ancestor sequence [29]. The BG505 gp120 monomer binds PG9, which is unusual given the quaternary nature of the PG9-Env interaction [29]. To make the BG505 SOSIP.664 gp140 construct, we introduced the following sequence changes (Fig. 1A): A501C and T605C (gp120-gp41_ECTO_ disulfide bond [5]); I559P in gp41_ECTO_ (trimer-stabilizing [6]); REKR to RRRRRR in gp120 (cleavage enhancement [31]); T332N in gp120 (introduction of epitopes dependent on glycan-332); stop codon at gp41_ECTO_ residue 664 (improvement of homogeneity and solubility [23], [24]). The codon-optimized gene for BG505 SOSIP.664 gp140 was obtained from Genscript (Piscataway, NJ) and cloned into pPPI4 using *PstI* and *NotI* [5].

Variants of the BG505 SOSIP.664 gp140 trimers bearing either a His-tag or a D7324 epitope-tag sequence at the C-terminus of gp41_ECTO_ were also made by adding the amino acid sequences GSGSGGSGHHHHHHHH or GSAPTKAKRRVVQREKR, respectively, after residue 664 in gp41_ECTO_ and preceding the stop codon. These proteins are designated SOSIP.664-His gp140 and SOSIP.664-D7324 gp140. We also made a His-tagged gp140 with the C501 and C605 cysteines replaced by their original residues, and with P559 similarly reverted to the original isoleucine (BG505 WT.664-His gp140). When expressed in the presence of excess furin to ensure efficient precursor cleavage, the absence of the SOS disulfide bond means the gp140 trimer is unstable and dissociates to gp120 and a trimeric form of His-tagged gp41_ECTO_ (BG505 gp41_ECTO_-His); the latter can be used in a NiNTA-capture enzyme-linked immunosorbent assay (ELISA; see below).

A monomeric BG505 gp120 with a similar sequence to the gp120 components of the gp140 trimers was designed by: introducing a stop codon into the SOSIP.664 construct at residue 512; reverting the optimized cleavage site to wild type (RRRRRR→REKR at residues 508–511); reverting the A501C change; introducing the D7324 epitope into the C5 region (R500K+G507Q); and making a L111A substitution to decrease gp120 dimer formation [29], [63]. A slightly modified version of BG505 gp120 that has been described previously [25] was used in DSC experiments. For this modification, the BG505 gp120 gene was cloned downstream of an IgK secretion signal in a phCMV3 plasmid and upstream of a His-tag. The cleavage site was mutated to prevent the His-tag from being cleaved off, leading to the following C-terminal sequence: RAKRRVVGSEKSGHHHHHH.

The BG505 gp160 clone for generating Env-pseudoviruses for neutralization assays has been described elsewhere [29]. We modified this clone by inserting the same T332N substitution that is present in the BG505 SOSIP.664 trimers, and refer to the resulting virus as BG505.T332N.

### Env protein expression

The Env proteins from various *env* genes described above were expressed in wild type, adherent HEK293T cells or the 293F variant that is adapted for suspension cultures, or in CHO-K1 cells, essentially as described [25], [27], [46], [64]. HEK293T and CHO-K1 cells were maintained in Dulbecco's modified Eagle's medium (DMEM) supplemented with 10% fetal calf serum (FCS), penicillin (100 U/ml), streptomycin (100 µg/ml), Glutamax (Invitrogen), non-essential amino acids (0.1 mM), sodium pyruvate (0.1 mM) and HEPES (0.1 mM). For gp140 trimer production, HEK293T or CHO-K1 cells were seeded at a density of 5.5×10^4^/ml in a Corning Hyperflask. After 3 days, when the cells had reached a density of 1.0×10^6^/ml, they were transfected using polyethyleneimine (PEI) as described elsewhere [65]. Briefly, PEI-MAX (1.0 mg/ml) in water was mixed with expression plasmids for Env and Furin [5] in OPTI-MEM. For one Corning Hyperflask, 600 µg of Env plasmid, 150 µg of Furin plasmid and 3 mg of PEI-MAX were added in 550 ml of growth media. Culture supernatants were harvested 72 h after transfection. BG505 gp120 used in differential scanning calorimetry (DSC) experiments was produced in HEK293F cells using a protocol similar to that previously described [25].

### Env protein purification

Env proteins were purified from the supernatants by affinity chromatography using either a 2G12 column or a *Galanthus nivalis* (GN)-lectin column [25], [27], [46], [64]. Briefly, transfection supernatants were vacuum filtered through 0.2-µm filters and then passed (0.5–1 ml/min flow rate) over the column. The 2G12 column was made from CNBr-activated Sepharose 4B beads (GE Healthcare) coupled to the bNAb 2G12 (Polymun Sciences, Klosterneuburg, Austria). Purification using this column was performed as follows: the beads were washed with 2 column volumes of buffer (0.5 M NaCl, 20 mM Tris, pH 8.0) before eluting bound Env proteins using 1 column volume of 3 M MgCl_2_. The eluted proteins were immediately buffer exchanged into 75 mM NaCl, 10 mM Tris, pH 8.0, using Snakeskin dialysis tubing (10K WCMO) (Thermo Scientific). The buffer-exchanged proteins were further concentrated using Vivaspin columns with a 30-kDa cut off (GE Healthcare). For GN-lectin affinity purification, the wash buffer was Dulbecco's phosphate buffer saline (DPBS) supplemented with 0.5 M NaCl was used, and elution was carried out using DPBS supplemented with 1 M methyl mannopyranoside.

In both cases, the affinity-purified Env proteins were further purified to size homogeneity using size exclusion chromatography (SEC) on a Superdex 200 26/60 column (GE Healthcare). A Superose 6 column was sometimes used for analytical or preparative purposes. The trimer fractions and, occasionally also the SOSIP gp140 monomer fractions, were collected and pooled. Protein concentrations were determined using either a bicinchonic acid-based assay (BCA assay; Thermo Scientific, Rockford, IL) or UV_280_ absorbance using theoretical extinction coefficients [66].

### SDS-PAGE and Blue Native-PAGE

Env proteins were analyzed using SDS-PAGE and BN-PAGE [6], [67] and stained using Coomassie blue or silver stain. The input material was mixed with loading dye and directly loaded onto a 4–12% Bis-Tris NuPAGE gel or a 10% Tris-Glycine gel (Invitrogen). The gels were run for 1.5 h at 200 V (0.07 A) using 50 mM MOPS, 50 mM Tris, pH 7.7 as the running buffer (Invitrogen).

### Differential scanning calorimetry (DSC)

Thermal denaturation was probed with a VP-DSC calorimeter (GE Healthcare). Before carrying out the experiments, all samples were extensively dialyzed against phosphate-buffered saline (PBS). The protein concentration was subsequently adjusted to 0.1–0.3 mg/ml, as described above. After loading the protein sample into the cell, thermal denaturation was probed at a scan rate of 90°C/h. Buffer correction, normalization and baseline subtraction procedures were applied before the data were analyzed using Origin 7.0 software. The data were fitted using a non-two-state model, as the asymmetry of some of the peaks suggested the presence of unfolding intermediates.

### Antibodies and Fabs

Antibody concentrations are generally recorded in ng/ml for neutralization assays and trimer binding ELISAs, but in nM for ITC and SPR experiments. Since the molecular mass of an average IgG molecule is approximately 150,000 Da, the conversion factors for IgG are: 1000 ng/ml = 6.7 nM and 1.0 nM = 150 ng/ml. For Fabs, the conversion factors are: 1000 ng/ml = 20 nM and 1.0 nM = 50 ng/ml.

MAbs were obtained as gifts, or purchased, from the following sources: John Mascola and Peter Kwong (VRC01, VRC03, VRC06, VRC06b, X5, F105); Dennis Burton (PGV04, PG9, PG16, PGT121–123, PGT125–128, PGT130, PGT135, PGT136, PGT145, b6, b12, F240); Polymun Scientific (447-52D, 2G12); Michel Nussenzweig (3BNC60, 3BNC117, 12A12, 45–46, 45–46W, 1NC9, 8ANC195, 3BC176, 3BC315); Michael Zwick (8K8, DN9); Barton Haynes (CH01, CH31, CH58, CH59, CH103, CH106, HG107, HG120); James Robinson (39F, 17b, 48d, 412d, A32, 19b, 14e, F91, 15e, 7B2). William Olson of Progenics Pharmaceuticals provided soluble CD4 (sCD4) and CD4-IgG2. The following reagents were obtained through the NIH AIDS Reagent Program, Division of AIDS, NIAID, NIH: D50 from Dr. Patricia Earl; 98-6 from Dr. Susan Zolla-Pazner; HJ16 from Dr. Antonio Lanzavecchia.

Fab PGT121, PGT128 and PGV04, as well as IgG 2G12 used in isothermal titration calorimetry (ITC) and electron microscopy (EM) experiments, were produced following a protocol similar to that previously described [25], [27]. Briefly, heavy and light chain genes were transfected in HEK293F cells using 293Fectin (Invitrogen). Secreted Fab or IgG were harvested 6–7 days post-transfection. The supernatant was directly loaded on either an anti-human λ light chain affinity matrix (CaptureSelect Fab λ; BAC) for PGT121 and PGT128 Fabs, an anti-human κ light chain affinity matrix (CaptureSelect Fab κ; BAC) for PGV04 Fab or on a Protein A column for 2G12 IgG. Elution was performed using a buffer containing 100 mM glycine, pH 2.7. The PGT121, PGT128 and PGV04 Fabs were subjected to MonoS (GE Healthcare) cation exchange chromatography to eliminate light chain dimers. All antibodies were subsequently purified to size homogeneity by gel filtration chromatography using a Superdex 200 column (GE Healthcare) in a buffer containing 150 mM NaCl, 20 mM Tris, pH 8.0.

The b6, F240, 14e and 19b Fab′ fragments used in EM and SPR experiments were produced by IgG digestion at 37°C for 1 h with the enzyme IdeS in a buffer containing 150 mM NaCl, 20 mM Bis-tris, pH 6.0. A reduction and alkylation reaction involving the addition of 10 mM dithiothreitol for 1 h, followed by 5 mM iodoacetamide, produced Fab′ from (Fab′)_2_. Subsequently, the Fab′ was purified away from the Fc fragment and undigested IgG using a Protein A affinity column.

### Neutralization assays

For Env-pseudovirus production, HEK293T cells (2×10^5^) were seeded at 2 ml per well in a 6-well tissue culture plate (Corning). After 1 d, the cells reached a confluence of 90–95%. Prior to transfection, the culture medium was refreshed using 2 ml of supplemented medium and the cells were transfected using Lipofectamine 2000 (Invitrogen). For one well, 1.6 µg of BG505.T332N plasmid and 2.4 µg of pSG3ΔEnv plasmid (obtained through the AIDS Research and Reference Reagent Program, Division of AIDS, NIAID, NIH from Drs. John C. Kappes and Xiaoyun Wu) were mixed in 250 µl of OPTI-MEM. A 10-µl aliquot of lipofectamine 2000 was mixed with 240 µl of OPTI-MEM immediately before addition to the solution containing the expression plasmids. After incubation for 20 min at room temperature, the mixture was added to the cells to initiate transfection. Culture supernatants were harvested 48 h later.

The TZM-bl reporter cell line, which stably expresses high levels of CD4 and the co-receptors CCR5 and CXCR4 and contains the luciferase and β-galactosidase genes under the control of the HIV-1 long-terminal-repeat promoter, was obtained through the NIH AIDS Research and Reference Reagent Program, Division of AIDS, NIAID, NIH (John C. Kappes, Xiaoyun Wu, and Tranzyme Inc. Durham, NC) [68], [69]. One day prior to infection, 1.7×10^4^ TZM-bl cells per well were plated on a 96-well plate in DMEM containing 10% FCS, 1× MEM nonessential amino acids, penicillin and streptomycin (both at 100 U/ml), and incubated at 37°C in an atmosphere containing 5% CO_2_ for 48 h. A fixed amount of virus (5 ng/ml of p24-antigen equivalent) was incubated for 30 min at room temperature with serial 1 in 3 dilutions of each test MAb [70], [71]. This mixture was added to the cells and 40 µg/ml DEAE, in a total volume of 200 µl. Two days later, the medium was removed. The cells were washed once with PBS (150 mM NaCl, 50 mM sodium phosphate, pH 7.0) and lysed in Reporter Lysis Buffer (Promega, Madison, WI). Luciferase activity was measured using a Luciferase Assay kit (Promega, Madison, WI) and a Glomax Luminometer according to the manufacturer's instructions (Turner BioSystems, Sunnyvale, CA). All infections were performed in duplicate. Uninfected cells were used to correct for background luciferase activity. The infectivity of each mutant without inhibitor was set at 100%. Nonlinear regression curves were determined and 50% inhibitory concentrations (IC_50_) were calculated using a sigmoid function in Prism software version 5.0.

### D7324-capture ELISA for monomeric and trimeric BG505 Env proteins

ELISAs were performed as described previously [33], [72], [73], with minor modifications. Microlon 96-well plates (Greiner Bio-One, Alphen aan den Rijn, The Netherlands) were coated overnight with Ab D7324 (Aalto Bioreagents, Dublin, Ireland) at 10 µg/ml in 0.1 M NaHCO_3_, pH 8.6 (100 µl/well). After washing and blocking steps, purified, D7324-tagged BG505 Env proteins were added at 100 ng/ml in TBS/10% FCS for 2 h. Unbound Env proteins were washed away, and TBS (150 mM NaCl, 20 mM Tris) plus 2% skimmed milk was added to further block non-specific protein-binding sites. Serially diluted MAbs or CD4-IgG2 in TBS/2% skimmed milk were then added for 2 h followed by 3 washes with TBS. In some cases, sCD4 (10 µg/ml) was added during the incubation with a test MAb. Horseradish peroxidase labeled goat-anti-human immunoglobulin G (IgG) (Jackson Immunoresearch, Suffolk, England) was added for 60 min at a 1∶3000 dilution (final concentration 0.33 µg/ml) in TBS/2% skimmed milk, followed by 5 washes with TBS/0.05% Tween-20. Colorimetric detection was performed using a solution containing 1% 3,3′,5,5′-tetramethylbenzidine (Sigma-Aldrich, Zwijndrecht, The Netherlands), 0.01% H_2_O_2_, 100 mM sodium acetate and 100 mM citric acid. Color development was stopped using 0.8 M H_2_SO_4_ when appropriate, and absorption was measured at 450 nm. In most experiments, SEC-purified BG505 gp120-D7324 or SOSIP.664-D7324 gp140 trimers (or sequence variants specified elsewhere) were used for the above assays. However, when specifically indicated, unpurified D7324-tagged BG505 SOSIP.664 gp140 (or mutants) were used instead.

### Ni-NTA-capture ELISA for BG505 Env proteins

Supernatants from cells transiently expressing SOSIP.664-His gp140 or gp41_ECTO_-His proteins were diluted 1∶2 in TBS/10% FCS and incubated for 2 h with Ni^2+^-nitrilotriacetic acid (Ni-NTA) coated Hissorb 96-well plates (Qiagen, Venlo, The Netherlands) [32], [33], [72], [73]. The subsequent procedures were exactly as described above for the D7324-capture ELISA.

### Surface plasmon resonance

MAb binding to trimers at 20°C was detected by two SPR-based methods using a Biacore 3000 instrument (GE Healthcare). In the first approach, His-tagged trimers were immobilized on Ni-NTA chips and the binding of solution-phase MAbs was recorded. After removing metallic contaminants via a pulse of EDTA (350 mM) in running buffer (150 mM NaCl, 10 mM Hepes, pH 7.4 plus 0.005% Tween20) for 1 min at a flow rate of 30 µl/min, the chip was loaded with Ni^2+^ by injecting NiCl_2_ (2.5 mM) for 1 min at a flow rate of 10 µl/min, resulting in a response of ∼50 RU. For all steps between the high EDTA pulses, the running buffer was supplemented with 50 µM EDTA. Purified SOSIP.664-His gp140 trimers (10,000 ng/ml) were injected at 10 µl/min for 2–3 min to capture the equivalent of ∼500 RU ( = R_L_). Control channels received neither trimer nor NiCl_2_. However, control cycles were performed by flowing the analyte over Ni^2+^-loaded NTA in the absence of trimer; there were no indications of non-specific binding. The analyte (IgG at 1,000 nM (150,000 ng/ml) or Fab at 500 nM (25,000 ng/ml)) was injected into the trimer sample and control channels at a flow rate of 50 µl/min. Association was recorded for 300 s, and dissociation for 600 s. After each cycle of interaction, the NTA-chip surface was regenerated with a pulse of EDTA (350 mM) for 1 min at a flow rate of 30 µl/min, followed by 3 washes with running buffer. A high flow rate of analyte solution (50 µl/min) was used to minimize mass-transport limitation; ln(dY/dX) plots for the association phase were linear with negative slopes, indicating that the binding was largely kinetically limited. Both control-channel and zero-analyte responses were subtracted.

In the second approach, Env-reactive MAbs were captured onto the chip by an immobilized anti-Fc Ab and the binding of solution-phase, untagged BG505 SOSIP.664 gp140 trimers was recorded. Affinity-purified goat anti-human IgG Fc (A80-104A, Bethyl Laboratories, Inc.) was diluted to 50 µg/ml in sodium acetate (pH 4.5) and then amine-coupled to dextran, reaching levels ∼10^4^ RU, in all four channels of CM5 chips. Env-reactive Abs were added (1 µg/ml in sodium acetate, pH 4.5) to three channels on each chip, at a flow rate of 5 µl/ml, and captured to response levels of 800–900 RU; the fourth channel served as a control surface. BG505 SOSIP.664 gp140 trimers at 200 nM (78,000 ng/ml) in running buffer (150 mM NaCl, 10 mM Hepes, pH 7.4, 3 mM EDTA plus 0.005% Tween20; note the higher EDTA concentration) were injected at a flow rate of 30 µl/min. Association was recorded for 300 s, and dissociation for 600 s.

### Isothermal titration calorimetry

ITC was performed using an Auto-iTC 200 instrument (GE Healthcare) using a protocol similar to one previously described [25], [27]. Briefly, prior to conducting the titrations, proteins were dialyzed against Tris-saline buffer (150 mM NaCl, 20 mM Tris, pH 8.0). Absorbance at 280 nm using calculated extinction coefficients served to determine and adjust protein concentrations [66]. The ligand present in the syringe was PGT121 Fab, PGT128 Fab or 2G12 IgG at concentrations ranging between 113 µM and 167 µM, while the BG505 SOSIP.664 trimer was present in the cell at a concentration of 4.3 µM. In each binding experiment, a 5 µcal reference power determination preceded the first injection of 0.5 µl, which was followed by 15 injections of 2.5 µl each at intervals of 180 s. Origin 7.0 software was used to derive the affinity constants (K_d_), the molar reaction enthalpy (ΔH) and the stoichiometry of binding (N), by fitting the integrated titration peaks via a single-site binding model (PGT121) or a two-site binding model (PGT128 and 2G12). All measured and derived thermodynamic parameters of binding are reported in Table 1.

### Electron microscopy

SEC-purified BG505 SOSIP.664 gp140 trimers, either alone or as Fab complexes (with b6, F240, 14e, 19b, PGV04, sCD4/17b), were analyzed by negative stain EM. A 3 µL aliquot containing ∼0.03 mg/mL of the trimer or Fab-trimer complex was applied for 5 s onto a carbon-coated 400 Cu mesh grid that had been glow discharged at 20 mA for 30 s, then negatively stained with Uranyl formate or Nano-W (Nanoprobes) for 30 s. Data were collected using a FEI Tecnai F20 or T12 electron microscope operating at 120 keV, with an electron dose of ∼55 e^−^/Å^2^ and a magnification of 52,000× that resulted in a pixel size of 2.05 Å at the specimen plane. Images were acquired with a Gatan US4000 CCD or Tietz TemCam-F416 CMOS camera using a nominal defocus range of 900 to 1300 nm.

### Image processing and 3D reconstruction

Particles were picked automatically using DoG Picker and put into a particle stack using the Appion software package [74], [75]. Initial, reference-free, two-dimensional (2D) class averages were calculated using particles binned by five via the Xmipp Clustering 2D Alignment [76] and sorted into classes. Particles corresponding to trimers or complexes were selected into a substack and binned by four before another round of reference-free alignment was carried out using the Xmipp Clustering and 2D alignment and IMAGIC software systems [77]. To analyze the interactions of the non-neutralizing Fabs (b6, F240, 14e, 19b) with BG505 SOSIP.664 gp140 trimers, the reference-free 2D class averages were examined. The Fabs were clearly visualized if they are bound to the trimer, allowing the percentage of bound trimers relative to unbound trimers to be tabulated.

For Fab-containing complexes, the unliganded trimer (EMDB 5019; [49]) was used as the initial model and refined against reference-free 2D class averages for 89 iterations without imposing symmetry. Fab densities were visible after 3 iterations. This model was then refined against raw particles for an additional 89 cycles with C3 symmetry imposed. For the unliganded BG505 SOSIP.664 gp140 trimer, an *ab initio* common lines model was calculated from reference-free 2D class averages in EMAN2 [78]. The final volumes for the EMDB 5019 trimer and BG505 SOSIP.664 gp140 trimer reconstructions were nearly identical. EMAN [79] was used for all 3D reconstructions. For the 3D average of BG505 SOSIP.664 with PGV04, 32,867 particles were included in the final reconstruction. For the 3D average of BG505 SOSIP.664 in complex with sCD4 and 17b, 22,145 particles were included in the final reconstruction. For the 3D average of unliganded BG505, 15,352 particles were included in the final reconstruction.

## Supporting Information

Figure S1 **Amino acid sequence of the BG505 SOSIP.664 protein.** Changes to the wt BG505 sequence are shown in bold and underlined.
(TIFF)

Figure S2 **Negative stain EM data of the unliganded BG505 SOSIP.664 gp140 trimer expressed in HEK293T cells.** (**A**) 2D class averages. (**B**) Fourier shell correlation (FSC) curve used to determine the ∼24 Å resolution of the final reconstruction.
(TIFF)

Figure S3 **Negative stain EM data of the BG505 SOSIP.664 gp140 trimer in complex with Fab PGV04.** (**A**) 2D class averages of trimer∶Fab complexes. (**B**) Fourier shell correlation (FSC) curve used to determine the ∼23 Å resolution of the final reconstruction.
(TIFF)

Figure S4 **Negative stain EM data of the BG505 SOSIP.664 gp140 trimer in complex with sCD4 and Fab 17b.** (**A**) 2D class averages of trimer∶sCD4∶Fab complexes. (**B**) Fourier shell correlation (FSC) curve used to determine the ∼22 Å resolution of the final reconstruction.
(TIFF)
