# Supplementary figures and images for: A Next-Generation Cleaved, Soluble HIV-1 Env Trimer, BG505 SOSIP.664 gp140, Expresses Multiple Epitopes for Broadly Neutralizing but Not Non-Neutralizing Antibodies
Source: PLoS Pathog. 2013 Sep 19;9(9):e1003618. doi: 10.1371/journal.ppat.1003618 (PMC3777863; doi:10.1371/journal.ppat.1003618)

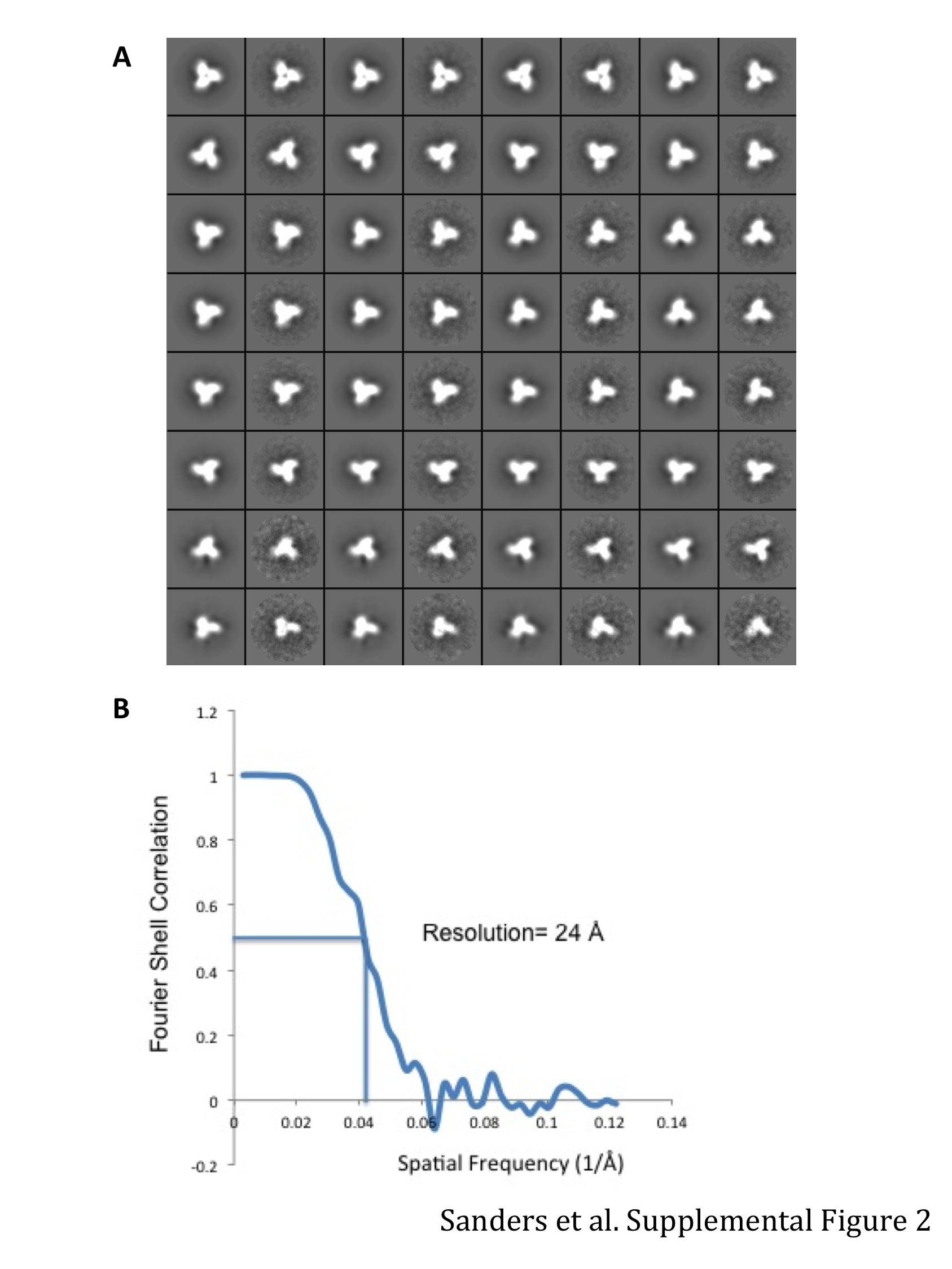

Supplement: Figure S2 — Negative stain EM data of the unliganded BG505 SOSIP.664 gp140 trimer expressed in HEK293T cells. (A) 2D class averages. (B) Fourier shell correlation (FSC) curve used to determine the ∼24 Å resolution of the final reconstruction. (TIFF) [file ppat.1003618.s002.tiff]

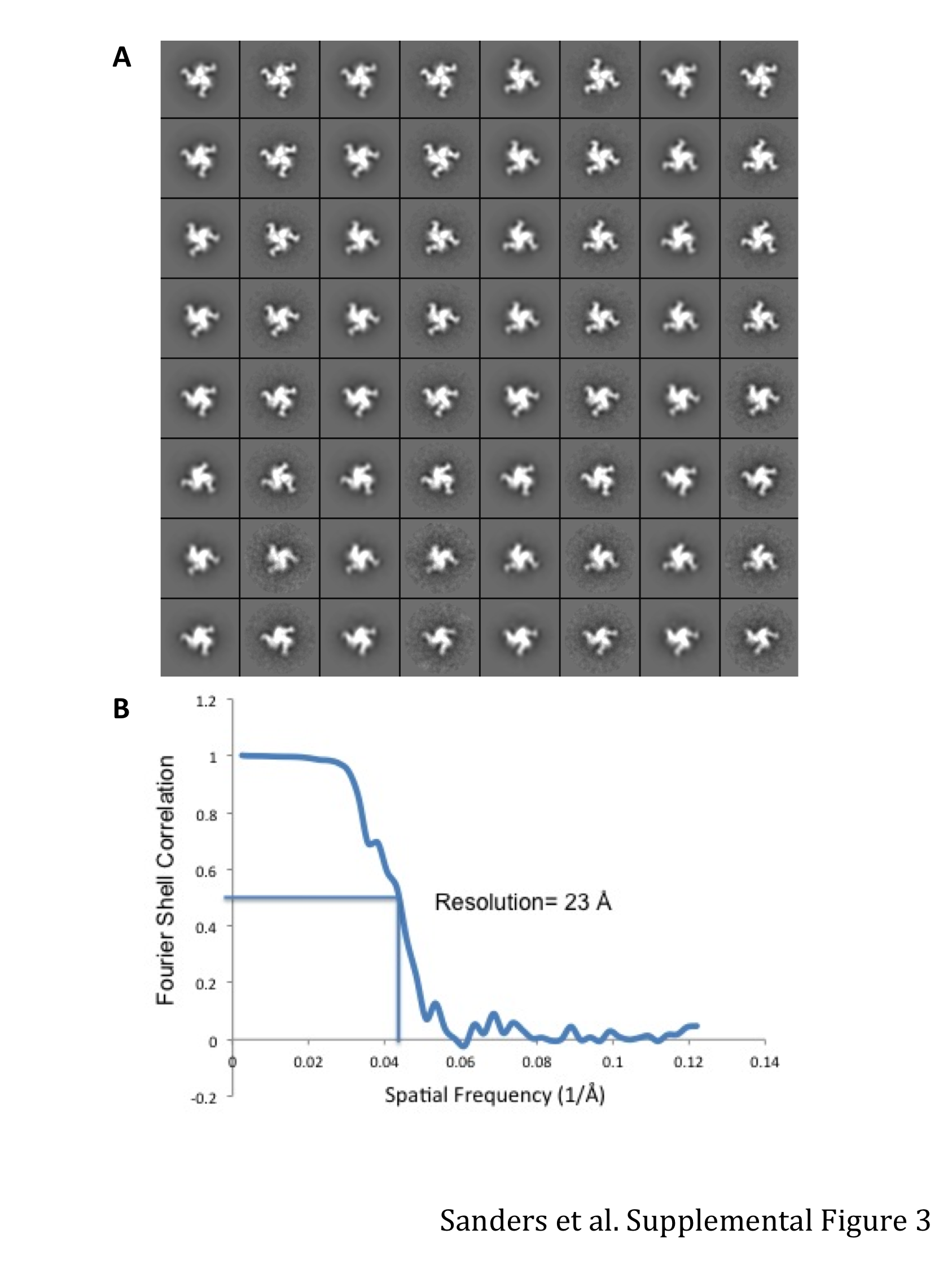

Supplement: Figure S3 — Negative stain EM data of the BG505 SOSIP.664 gp140 trimer in complex with Fab PGV04. (A) 2D class averages of trimer∶Fab complexes. (B) Fourier shell correlation (FSC) curve used to determine the ∼23 Å resolution of the final reconstruction. (TIFF) [file ppat.1003618.s003.tiff]

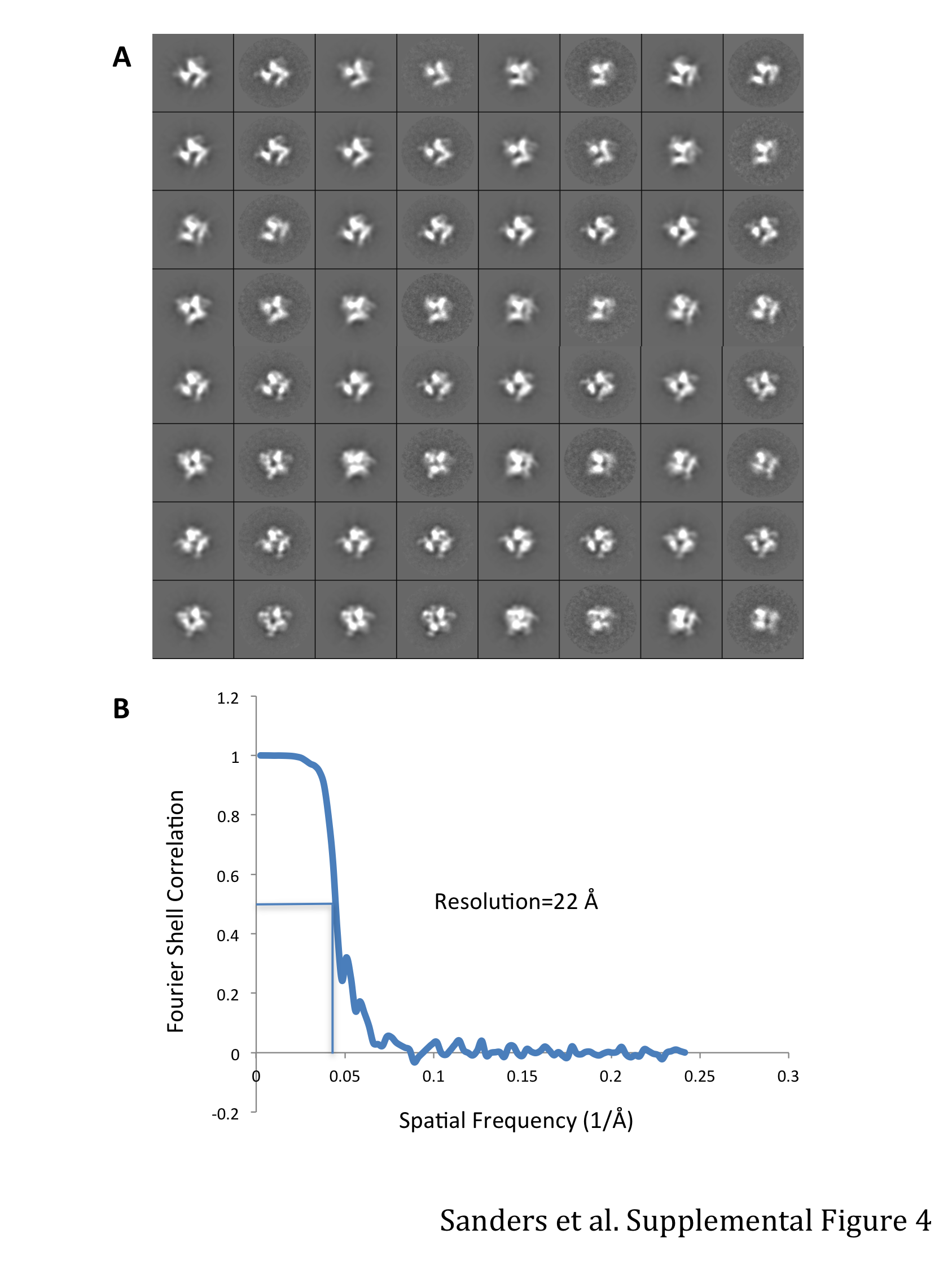

Supplement: Figure S4 — Negative stain EM data of the BG505 SOSIP.664 gp140 trimer in complex with sCD4 and Fab 17b. (A) 2D class averages of trimer∶sCD4∶Fab complexes. (B) Fourier shell correlation (FSC) curve used to determine the ∼22 Å resolution of the final reconstruction. (TIFF) [file ppat.1003618.s004.tiff]
